# PSMD14 Stabilizes SLC7A11 to Ameliorate Glucocorticoid‐Induced Osteoporosis by Suppressing Osteocyte Ferroptosis

**DOI:** 10.1002/advs.202414902

**Published:** 2025-05-30

**Authors:** Yifeng Shi, Qian Tang, Sunren Sheng, Hongyi Jiang, Chen Jin, Chencheng Zhou, Chenglong Xie, Lin Zheng, Di Zhang, Hui Xu, Cong Xu, Haiwei Ma, Guangheng Xiang, Wenfei Ni, Xiaoyun Pan, Lei Yang, Huazi Xu, Yu Qian, Aimin Wu, Xiangyang Wang, Gang Zheng

**Affiliations:** ^1^ Key Laboratory of Orthopaedics of Zhejiang Province Department of Orthopedics The Second Affiliated Hospital and Yuying Children's Hospital of Wenzhou Medical University Wenzhou 325000 China; ^2^ Department of Orthopedic Surgery Shanghai Jiao Tong University Affiliated Shanghai Sixth People's Hospital Shanghai 200233 China; ^3^ Department of Orthopaedics and Traumatology The First Affiliated Hospital of Zhejiang Chinese Medical University (Zhejiang Provincial Hospital of Chinese Medicine) Hangzhou 310006 China; ^4^ Department of Orthopaedics Surgery Lishui Central Hospital and Fifth Affiliated Hospital of Wenzhou Medical University Lishui 323000 China

**Keywords:** ferroptosis, glucocorticoid‐induced osteoporosis, PSMD14, SLC7A11, ubiquitination

## Abstract

Glucocorticoid‐induced osteoporosis (GIOP) remains the most prevalent complication compromising bone health in patients undergoing glucocorticoid (GC) therapy. Despite its clinical significance, osteocyte death, a pivotal initiator of GC‐driven bone metabolic imbalance, has received insufficient attention. This study identifies ferroptosis, an iron‐dependent regulated cell death mechanism, as a novel pathological phenotype of osteocytes in GC microenvironments. Utilizing GPX4 conditional knockout mice and pharmacological ferroptosis inhibitors, this work demonstrates that osteocyte ferroptosis exacerbates GIOP progression. Metabolomic profiling reveals cystine insufficiency and glutathione depletion in GC‐treated osteocytes. Mechanistically, GCs directly impede the deubiquitinase PSMD14 from binding to SLC7A11, thereby promoting SLC7A11 ubiquitination and proteasomal degradation, which sharply diminishes cystine uptake. Bone‐targeting adeno‐associated virus‐mediated PSMD14 overexpression stabilized SLC7A11, attenuating both osteocytic ferroptosis and bone loss in GIOP mice. Through high‐throughput virtual screening, this work identifies Pantethine as a potent PSMD14 activator that enhances deubiquitinase activity, restores SLC7A11 expression in osteocytes, and mitigates osteoporosis. Collectively, this study elucidates the role and mechanism of osteocyte ferroptosis in GIOP pathogenesis and proposes PSMD14‐targeted therapy as a viable clinical strategy.

## Graphical abstract

Glucocorticoid‐induced osteoporosis (GIOP) triggers osteocyte ferroptosis via SLC7A11 degradation. PSMD14 stabilizes SLC7A11 by counteracting glucocorticoid‐driven ubiquitination, preserving cystine uptake and glutathione synthesis. AAV‐mediated PSMD14 delivery or its agonist Pantethine rescues osteocyte survival and bone loss in GIOP mice. This study identifies ferroptosis as a key mechanism in GIOP and proposes PSMD14‐targeted therapy for clinical translation.

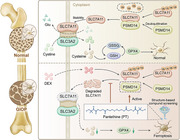

## 1 Introduction

Glucocorticoid‐induced osteoporosis (GIOP), a prominent cause of fragility fractures in glucocorticoid (GC)‐treated patients, is recognized as a musculoskeletal complication of long‐term GC use.^[^ ^1^ ^]^ Despite their widespread clinical application, GCs lack effective alternatives due to their indispensable anti‐inflammatory and immunosuppressive properties. Epidemiological studies reveal that approximately 1% of adults in the United States and the United Kingdom receive long‐term oral GC therapy, with prevalence rising to 3% among elderly populations.^[^ ^2^, ^3^ ^]^ A multicenter survey in China further demonstrated that over 80% of GC‐treated patients experience bone loss, of whom 41.4% progress to osteoporosis.^[^ ^4^ ^]^ These findings underscore the significant challenges GIOP poses to patients’ daily lives and primary disease management.

Current evidence highlights that GC‐mediated bone loss is classically characterized by a metabolic imbalance marked by excessive bone resorption and suppressed bone formation.^[^ ^1^, ^5^ ^]^ Although therapeutic strategies targeting osteoclasts or osteoblasts show partial efficacy, they remain insufficient to address the multifaceted pathology of GIOP. The potential mechanisms and roles of osteocytes, the most abundant cells in mineralized bone and distributed within the cortex and trabeculae, are still under‐appreciated.^[^ ^6^ ^]^ These cells orchestrate metabolic homeostasis through the lacunar‐canalicular network, secreting signaling molecules to coordinate osteoblast and osteoclast activity. Recent studies indicate that GC exposure induces osteocyte apoptosis, disrupts the lacunar‐canalicular architecture, and dysregulates osteocyte‐derived factors such as DKK1 and sclerostin.^[^ ^7^, ^8^, ^9^ ^]^ However, the precise mechanisms underlying GC‐induced osteocyte dysfunction remain elusive.

Ferroptosis is a new type of programmed cell death associated with oxidative disorder, accompanied by unique pathological events such as decompensation of the glutathione‐glutathione peroxidase 4 (GSH‐GPX4) system, accumulation of lipid peroxides, and iron overload‐mediated Fenton reaction.^[^ ^10^ ^]^ In the context of GC‐induced osteonecrosis of the femoral head (ONFH), transcriptomic analyses of patient serum have identified ferroptosis‐related gene signatures, while in vitro and in vivo models demonstrate GC‐triggered ferroptosis in mesenchymal stem cells.^[^ ^11^, ^12^, ^13^ ^]^ These observations suggest ferroptosis may contribute to GIOP pathogenesis. Nevertheless, the interplay between osteocytes and ferroptosis in GC microenvironments is poorly understood. Existing studies predominantly focus on GC‐mediated oxidative stress, leaving upstream triggers and osteocyte‐specific ferroptosis mechanisms unexplored.^[^ ^13^, ^14^ ^]^

In this study, based on human, mouse, and MLO‐Y4 cell samples, we multi‐dimensionally confirmed that ferroptosis occurred in osteocytes during GIOP progression, accelerating bone mineral loss. Regarding the priming mechanism, GC‐induced SLC7A11 degradation disrupts cystine/glutathione metabolism in osteocytes, precipitating lipid peroxidation. Molecularly, diminished interaction with the deubiquitinase PSMD14 underlies SLC7A11 downregulation. Genetic overexpression of PSMD14 conferred protection against osteocyte ferroptosis and preserved bone mass in GC‐exposed mice. Through virtual screening and biological validation, we identified antethine (PT) from a library of >70 000 compounds as a PSMD14 activator with anti‐ferroptotic and anti‐GIOP efficacy. Collectively, our findings elucidate novel mechanisms of GC‐induced osteocyte injury and propose PSMD14 as a therapeutic target for GIOP management.

## 2 Results

### 2.1 The Glucocorticoid Stimulates Osteocyte Ferroptosis In Vivo and In Vitro

**Figure 1:**
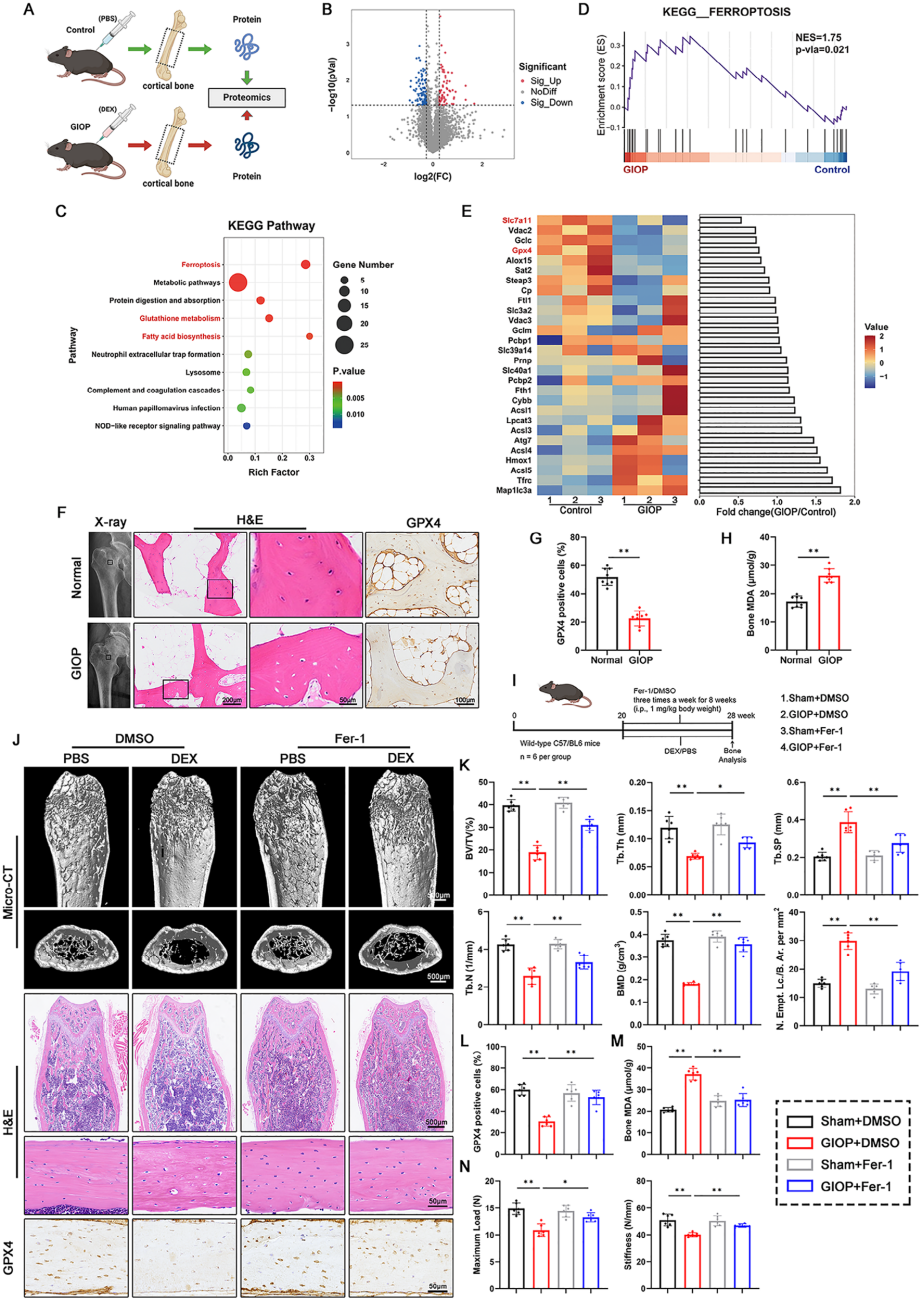
The glucocorticoid microenvironment induced osteocyte ferroptosis in vivo. A) Schematic illustration of the preparation of cortical bone samples for proteomic analysis. 20‐week‐old wild‐type C57BL/6 mice were subcutaneously injected with 2.5 mg kg^−1^ DEX (or PBS as control) three times a week for 8 weeks to establish the GIOP (or control) group. B) Volcano plot showing differentially expressed proteins from proteomics. C) KEGG analysis of the differentially expressed proteins. D) GSEA enrichment plot evaluating the changes of ferroptosis‐related proteins. E) Heatmap of representative proteins related to ferroptosis pathway between the Control and GIOP groups of mouse cortical bone. F) X‐ray imaging, H&E staining, and GPX4 histochemical staining of femoral tissues from normal and GIOP patients. G) Quantitative analysis of GPX4‐positive osteocytes in human femoral based on IHC staining (n = 8 per group). H) MDA content in human femoral tissue (n = 8 per group). I) Schematic showing the experimental protocol for 8‐weeks of DMSO/Fer‐1 injections in mice. J) Micro‐CT 3D restruction, H&E staining, and GPX4 histochemical staining of the distal femur of mice in each group. The processing details of each group are shown in (I). K) Distal femur BV/TV, Tb.Th, Tb.Sp, Tb.N, and BMD of mice in each group were measured by micro‐CT (n = 6 per group). Quantitative analysis of the empty lacunae in cortical bone (Number of empty lacunae with respect to bone area, N. Empt. Lc./B. Ar. per mm^2^) based on H&E staining (n = 6 per group). L) Quantification of GPX4‐positive osteocytes in mouse cortical femurs based on IHC staining (n = 6 per group). M) MDA content in tibia tissue of mice in each group (n = 6 per group). N) Maximum load and Maximum deflection of femoral cortical bone evaluated by the three‐point bending test (n = 6 per group). Data are expressed as mean ± SD, with biologically individual data points shown. *P* values were determined by unpaired two‐tailed Student's *t* test (G,H) and two‐way ANOVA test with Tukey's multiple comparisons (K–N), * *p* <  0.05, ** *p* <  0.01.

To investigate osteocyte status in GIOP pathogenesis, we established an in vivo model through subcutaneous dexamethasone (DEX, a potent and long‐acting synthetic glucocorticoid) administration in mice. Femoral radiography and histomorphometric analysis revealed that DEX treatment significantly reduced bone mass and disrupted trabecular architecture, confirming successful GIOP model establishment (**Figure** **1**J, K). And we conducted three‐point bending tests on the femoral cortex to simulate clinical fracture occurrence, recording parameters such as load and stiffness to evaluate the mechanical strength of femoral cortex. The results showed significant changes in maximum load and stiffness, indicating that DEX destroyed the mechanical strengthen of cortical bone (Figure 1N). To minimize confounding effects from other bone marrow cells, mid‐femoral cortical bone was isolated for proteomic analysis (Figure 1A; Table S1, Supporting Information). A total of 163 differentially expressed proteins were identified, of which 66 proteins were upregulated and 97 proteins were downregulated (Figure 1B). KEGG pathway analysis highlighted significant enrichment in lipid metabolism and ferroptosis‐related pathways (Figure 1C), while GSEA confirmed ferroptosis pathway activation in the GIOP group (Figure 1D). A heatmap visualized key ferroptosis‐associated protein alterations (Figure 1E). To illustrate the association between ferroptosis and glucocorticoids, we compared levels of ferroptosis hallmarks in the femoral neck region of patients undergoing hip replacement. GPX4, as a peroxidase, could utilize GSH to convert lipid hydroperoxides into nontoxic lipid alcohols to protect the plasma membrane. Immunohistochemical (IHC) staining indicated that the number of osteocytes expressing GPX4 in trabecular bone decreased after DEX injection (Figure 1F,G). Malondialdehyde (MDA) levels in the human femur, the end product of lipid oxidation, were also elevated in GIOP patients (Figure 1H). Similarly, DEX‐treated mice exhibited osteocyte GPX4 depletion and increased bone MDA content (Figure 1L,M), corroborating ferroptosis involvement.

Based on these findings, we determined that ferroptosis significantly contributes to the development of GIOP. To further validate this observation, MLO‐Y4 cells were treated with DEX in the presence of an iron chelator (desferrioxamine, DFO) and a lipophilic antioxidant (ferrostatin‐1, Fer‐1). Specifically, CCK‐8 assays demonstrated DEX‐induced cytotoxicity in dose‐ and time‐dependent manners (Figure S1A, Supporting Information), reversible by Fer‐1 or DFO co‐treatment (Figure S1B,C, Supporting Information). To further confirm the occurrence of ferroptosis, we performed GPX4 assays, MDA detection and C11‐BODIPY staining in DEX‐exposed MLO‐Y4 cell with or without ferroptosis inhibitors. DEX‐mediated GPX4 depletion was reversed by Fer‐1 or DFO (Figure S1D,E, Supporting Information). C11‐BODIPY probes and MDA quantification intuitively reflected the intensity of intracellular lipid peroxidation, and the results indicated that Fer‐1 or DFO reduced lipid peroxides and MDA content (Figure S1F–H, Supporting Information).

Additionally, the impact of Fer‐1 on the progression of GIOP was assessed using radiographic imaging, histological analysis, and biomechanical testing (Figure 1I–N). Micro‐CT reconstruction visualized the bone microarchitecture of distal femur in mice (Figure 1J). Compared with mice injected with DEX alone, Fer‐1‐treated mice exhibited richer bone mass, higher bone mineral density, and denser trabecular bone (Figure 1K). In the H&E staining, the lacunae of osteocytes enlarged and even lost their nuclei, leaving only cavity in GIOP femurs (Figure 1J,K). IHC results suggested that GPX4 expression in osteocytes decreased after DEX treatment, and the MDA level in tibia was elevated in GIOP mice. As expected, Fer‐1 application rescued osteocyte morphology, reduced intracortical lacunae, increased GPX4 expression, and reduced MDA content, highlighting the importance of ferroptosis in GIOP progression (Figure 1J,L,M). Moreover, three‐point bending test showed that the femoral cortex of GIOP mice treated with Fer‐1 showed greater ultimate load and stiffness (Figure 1N). In conclusion, inhibiting ferroptosis can be a viable strategy against GIOP.

### 2.2 Osteocyte Ferroptosis Involves in Glucocorticoid‐Induced Bone Loss and Destruction

**Figure 2:**
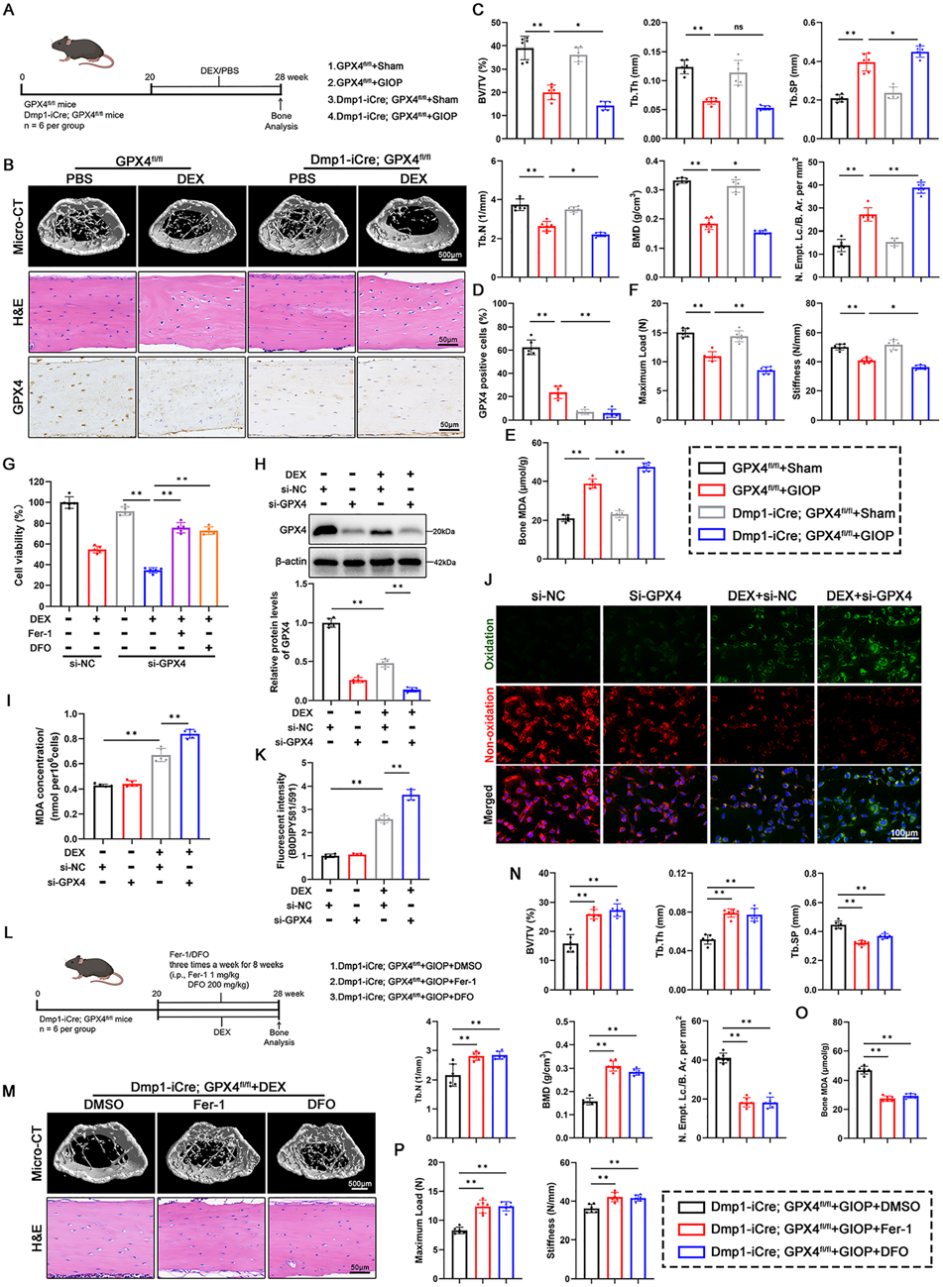
Knockout of GPX4 in osteocytes triggered ferroptosis and bone loss upon DEX exposure. A) Schematic showing the experimental protocol for 8‐weeks of DEX injections in transgenic mice. Control: GPX4^fl/fl^ mice. cKO (conditional osteocytic GPX4 knockout): Dmp1‐Cre; GPX4^fl/fl^ mice. B) Micro‐CT 3D restruction, H&E staining, and GPX4 histochemical staining of the distal femur of mice in each group. The processing details of each group are shown in (A). C) Distal femur BV/TV, Tb.Th, Tb.Sp, Tb.N, and BMD of mice in each group were measured by micro‐CT (n = 6 per group). Quantitative analysis of the empty lacunae in cortical bone (Number of empty lacunae with respect to bone area, N. Empt. Lc./B. Ar. per mm^2^) based on H&E staining (n = 6 per group). D) Quantification of GPX4‐positive osteocytes in mouse cortical femurs based on IHC staining (n = 6 per group). E) MDA content in mouse tibia tissue (n = 6 per group). F) Maximum load and Maximum deflection of femoral cortical bone evaluated by the three‐point bending test (n = 6 per group). G) CCK‐8 assay was performed on siRNA‐transfected MLO‐Y4 cells (50 nM si‐NC or si‐GPX4 for 24 h). After transfection was completed, MLO‐Y4 cells were treated with DEX (100 µM) for 48 h while adding the ferroptosis inhibitors (Fer‐1 1µM or DFO 100µM) (n = 5 per group). H–K) Western blot and quantitative analysis of GPX4 protein (H), MDA concentration detection (I), C11‐BODIPY 581/591 staining (J) and quantitative analysis (K) were performed on siRNA‐transfected MLO‐Y4 cells (50 nM si‐NC or si‐GPX4 for 24 h). After transfection was completed, MLO‐Y4 cells were treated with PBS or DEX (100 µM) for 48 h (n = 5 per group). L) Schematic showing the experimental protocol for 8‐weeks of Fer‐1/DFO injections in GIOP transgenic mice. M) Micro‐CT 3D restruction and H&E staining of the distal femur of mice in each group. The processing details of each group are shown in (K). N) Distal femur BV/TV, Tb.Th, Tb.Sp, Tb.N, and BMD of mice in each group were measured by micro‐CT (n = 6 per group). Quantitative analysis of the empty lacunae in cortical bone (Number of empty lacunae with respect to bone area, N. Empt. Lc./B. Ar. per mm^2^) based on H&E staining (n = 6 per group). O) MDA content in tibia tissue of mice in each group (n = 6 per group). P) Maximum load and Maximum deflection of femoral cortical bone evaluated by the three‐point bending test (n = 6 per group). Data are expressed as mean ± SD, with biologically individual data points shown. *p* values were determined by one‐way ANOVA test with Tukey's multiple comparisons (G–I,K,N–P) and two‐way ANOVA test with Tukey's multiple comparisons (C–F), ns, *p* > 0.05, * *p* <  0.05, ** *p* <  0.01.

To reveal the contribution of osteocyte ferroptosis to GIOP, we crossed GPX4^fl/fl^ mice with Dmp1‐iCre mice to obtain osteocytic GPX4‐deficient mice. The detailed construction strategy and genotyping verification of GPX4cKO (Dmp1‐iCre; GPX4^fl/fl^) mice were shown in Figure S2A,B, Supporting Information. GPX4cKO mice exhibited normal bone and cartilage development (Figure S2C–E, Supporting Information). We administered DEX to transgenic mice and subsequently assessed osteoporosis and ferroptosis‐related indicators after 8 weeks (**Figure** **2**A). Micro‐CT analysis found that, under DEX exposure, BMD, BV/TV, Tb.Th and Tb.N of GPX4cKO mice were significantly reduced, while Tb.Sp was increased compared with the control group (GPX4^fl/fl^ mice) (Figure 2B,C). Histological evaluation via H&E staining, MDA quantification, and mechanical testing indicated that GPX4 deletion in osteocytes exacerbated the formation of cortical lacunae, lipid peroxidation end products, and femoral cortical fragility (Figure 2B–F). Next, we investigated whether ferroptosis inhibitors (Fer‐1 or DFO) could rescue DEX‐treated GPX4cKO mice. The experimental protocol is depicted in Figure 2L. As anticipated, Fer‐1 and DFO effectively mitigated bone loss, intracortical destruction, lipid peroxidation, and cortical fragility in GPX4cKO mice (Figure 2M–P). Collectively, these findings suggest that osteocytic ferroptosis is a novel pathological event in GIOP, accelerating bone loss.

To further explore the role of GPX4 in GIOP osteocytes, we established GPX4 knockdown and overexpression MLO‐Y4 cell lines. The transfection efficiency of GPX4 was shown in Figure S3A,C, Supporting Information. CCK8 assay revealed that inhibiting GPX4 expression significantly exacerbated DEX‐induced cell death in MLO‐Y4 cells. Conversely, treatment with Fer‐1 and DFO effectively mitigated both GPX4 inhibition and DEX‐induced cell death (Figure 2G). Under physiological conditions, GPX4 knockdown did not affect lipid peroxidation in MLO‐Y4 cells but exacerbated DEX‐induced ferroptosis, as evidenced by increased MDA production and lipid peroxide (Figure 2H–K). Unsurprisingly, in MLO‐Y4 cells overexpressing GPX4, DEX‐mediated cell death, GPX4 depletion and lipid peroxidation reactions were restricted to varying degrees (Figure S3B–F, Supporting Information). These data confirm that GPX4 loss potentiates osteocyte ferroptosis under glucocorticoid stress.

### 2.3 SLC7A11 Degradation is Essential for Glucocorticoid‐Triggered Ferroptosis in Osteocytes

**Figure 3:**
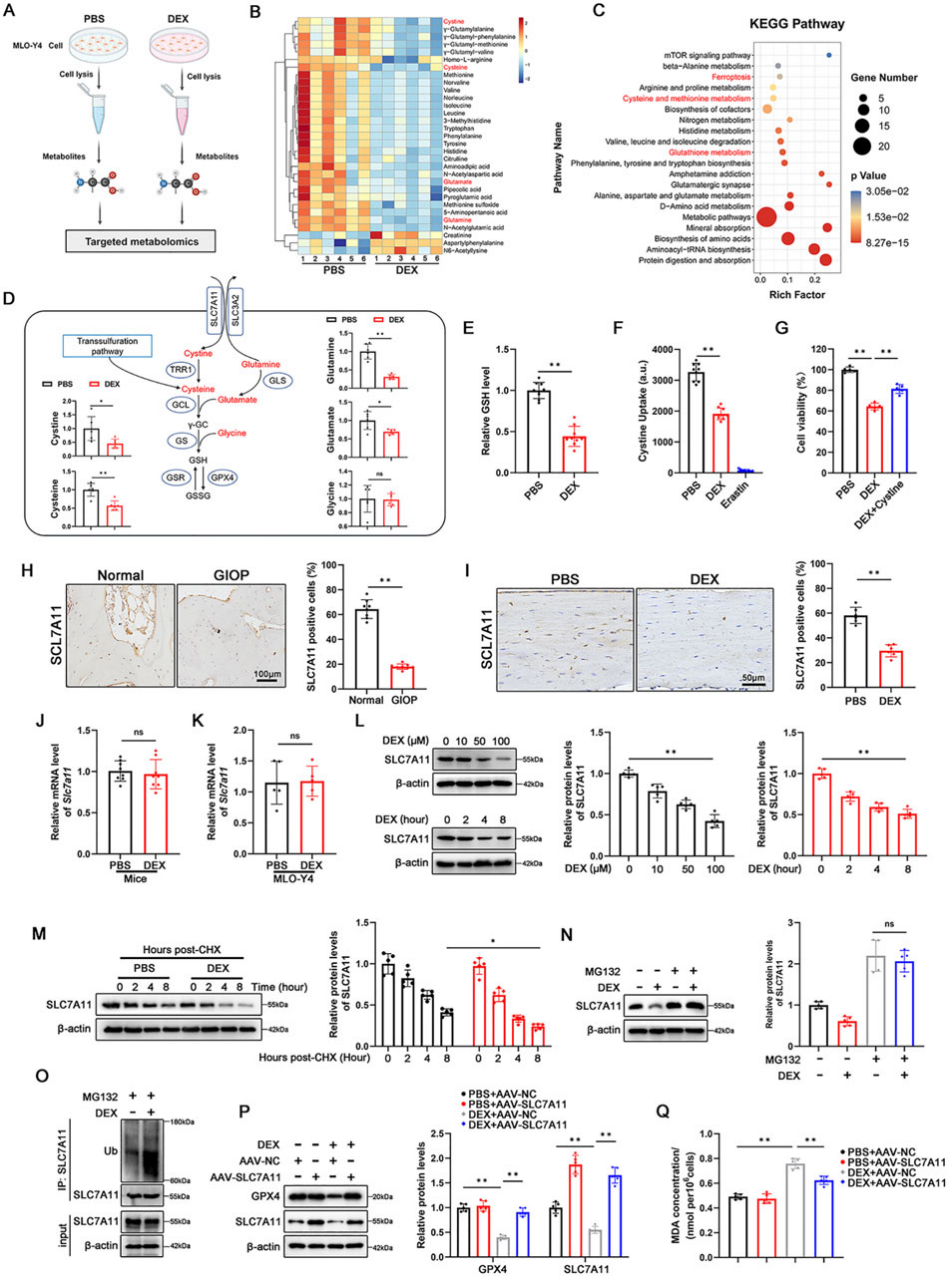
DEX accelerated UPS‐dependent degradation of SLC7A11 and restricted cystine/glutathione metabolism. A) Schematic illustration of the preparation of cell samples for targeted metabolomics. MLO‐Y4 cells were treated with PBS or DEX (100 µM) for 48 h. B) Heatmap showing significantly differently expressed metabolites (*p* < 0.05) between PBS and DEX groups. C) KEGG analysis of the differentially expressed metabolites. D) Illustration of the cystine/glutathione metabolism pathway and the relative levels of cystine, cysteine, glutamine, glutamate, and glycine (n = 6 per group). E) GSH level detection was performed on MLO‐Y4 cells treated with PBS or DEX (100 µM) for 48 h (n = 9 per group). F) Cystine uptake assay was performed on MLO‐Y4 cells treated with PBS, DEX (100 µM) or Erastin (10 µM, for positive control) for 48 h (n = 9 per group). G) CCK‐8 assay was performed on MLO‐Y4 cells treated with DEX (100 µM) and Cystine (100 µM) for 48 h (n = 5 per group). H) Histochemical staining and quantitative analysis of SLC7A11 in human femoral from normal and GIOP patients (n = 8 per group). I) Histochemical staining and quantitative analysis of SLC7A11 in the distal cortical femurs of mice treated with PBS or DEX (n = 6 per group). J) *Slc7a11* mRNA levels in femurs of mice treated with PBS or DEX (n = 6 per group). K) *Slc7a11* mRNA levels in MLO‐Y4 cell treated with PBS or DEX (100 µM) for 48 h (n = 5 per group). L) Western blot and quantitative analysis of SLC7A11 protein was performed on MLO‐Y4 cells treated with different doses of DEX (0, 10, 50, 100 µM) for 8 h (Top). Western blot and quantitative analysis of SLC7A11 protein was performed on MLO‐Y4 cells treated with DEX (100 µM) for different time periods (0, 2, 4, 8 h) (Bottom) (n = 5 per group). M) Western blot and quantitative analysis of SLC7A11 protein were performed on MLO‐Y4 cells treated with CHX (50 µg mL^−1^) and PBS/DEX (100 µM) for the indicared time (0, 2, 4, 8 h) (n = 5 per group). CHX: cycloheximide. N) Western blot and quantitative analysis of SLC7A11 protein were performed on MLO‐Y4 cells treated with MG132 (10 µM) with or without DEX (100 µM) for 8 h (n = 5 per group). O) Immunoprecipitation of SLC7A11 ubiquitination were performed on 10 µM MG132‐treated MLO‐Y4 cells supplemented with or without DEX (100 µM) for 8 h. P,Q) Western blot of GPX4 and SLC7A11 proteins (P) and MDA concentration detection (Q) were performed on AAV‐transfected MLO‐Y4 cells. After AAV‐NC/SLC7A11 transfection was completed, MLO‐Y4 cells were treated with PBS or DEX (100 µM) for 8 h (n = 5 per group). AAV: adeno‐associated virus. Data are expressed as mean ± SD, with biologically individual data points shown. *p* values were determined by unpaired two‐tailed Student's *t* test (D–F,H–K), one‐way ANOVA test with Tukey's multiple comparisons (G,L) and two‐way ANOVA test with Tukey's multiple comparisons (M,N,P,Q), ns, *p* > 0.05, * *p* <  0.05, ** *p* <  0.01.

As the core gatekeeper of ferroptosis, GPX4's detoxification originates from the reduced glutathione, whose rapid consumption without replenishment inhibits the activity and expression of GPX4.^[^ ^15^ ^]^ Based on previous proteomics, KEGG analysis revealed that the glutathione metabolism pathway was significantly affected (Figure 1C). Amino acid metabolomics was performed to dissect the intrinsic mechanisms (**Figure** **3**A; Table S2, Supporting Information). Heatmap analysis showed that critical intermediates of glutathione metabolism in MLO‐Y4 cells were significantly downregulated after DEX stimulation (Figure 3B). KEGG analysis of differential metabolites focused on glutathione metabolism and ferroptosis pathways (Figure 3C). Cystine, the rate‐limiting precursor for glutathione synthesis, is imported from the external environment by the cystine/glutamate antiporter system (system Xc⁻) on the plasma membrane and is rapidly converted to cysteine within the cytoplasm (Figure 3D). After DEX treatment, crucial metabolites, including cystine, cysteine, glutamate, and glutamine, declined, while glycine levels remained stable (Figure 3D). The final product, GSH content, decreased upon DEX stimulation (Figure 3E). Quantitatively, DEX‐treated MLO‐Y4 cells exhibited lower cystine uptake than the phosphate‐buffered saline (PBS) group, consistent with the effect of Erastin, a system Xc⁻ dependent ferroptosis agonist (Figure 3F). Exogenous cystine supplementation partially reversed DEX‐induced osteocytic death, MDA production, and lipid peroxidation (Figure 3G; Figure S4, Supporting Information).

Given the observed cystine uptake defects, we hypothesized that SLC7A11, the catalytic subunit of system Xc⁻, dominates this biological process within the context of GIOP. Femoral IHC staining suggested that DEX downregulated SLC7A11 expression in human and mouse samples (Figure 3H,I). Western blot analysis showed that SLC7A11 expression in MLO‐Y4 cells decreased in a time‐ and dose‐dependent manner upon DEX treatment, while mRNA transcription remained unaffected in both animal and cell experiments, indicating the importance of post‐transcriptional degradation (Figure 3J–L). In MLO‐Y4 cells, cycloheximide (CHX) was used to selectively inhibit protein synthesis, and a reduced half‐life of the SLC7A11 protein was detected following DEX treatment (Figure 3M). However, DEX‐induced SLC7A11 loss was counteracted by the proteasome inhibitor MG132 (Figure 3N). We next examined the effect of DEX on SLC7A11 ubiquitination. As shown in Figure 3O, DEX significantly increased the ubiquitinated form of SLC7A11 in MG132‐treated MLO‐Y4 cells. Collectively, these results indicate the primary role of the ubiquitin‐proteasome system (UPS) in the reduction of SLC7A11 following DEX exposure.

Although SLC7A11 is a recognized ferroptosis inhibitory molecule, its relationship with ferroptosis in DEX exposure still requires to be established before elucidating the details of ubiquitination. Overexpression of SLC7A11 by AAV reversed DEX‐mediated ferroptosis events such as the decrease in GPX4 and the increase in MDA (Figure 3P,Q).

### 2.4 PSMD14 Interacts with SLC7A11 in Response to DEX

**Figure 4:**
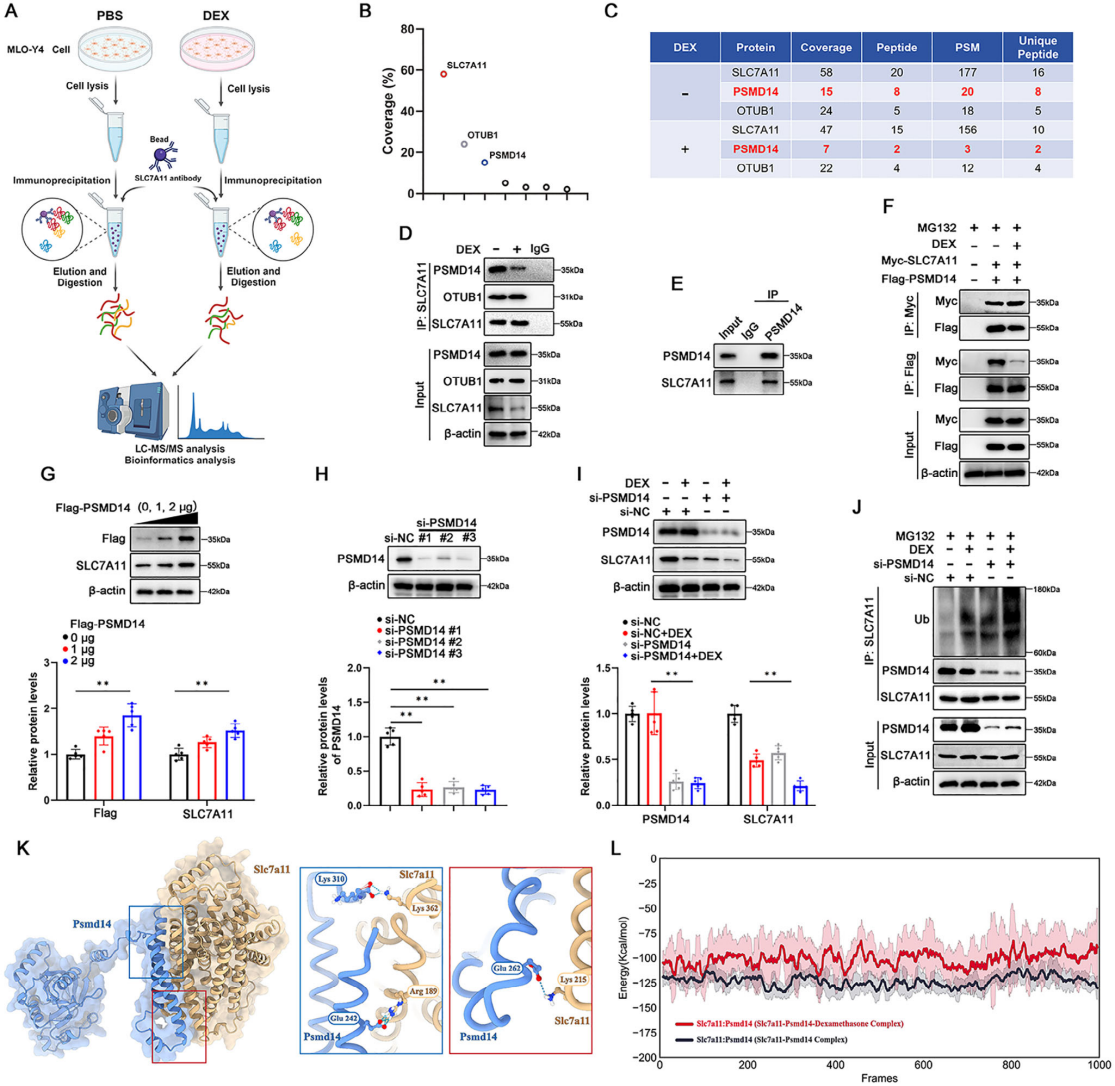
PSMD14 acts as a major regulator for SLC7A11 stability in response to DEX stimulation. A) Schematic illustration of the preparation of SLC7A11‐binding protein sample in MLO‐Y4 cells for LC‐MS/MS analysis to identify the deubiquitinase. B) After proofreading using the UbiBrowser library, the SLC7A11‐bound deubiquitinases were ranked according to sequence coverage. C) LC‐MS/MS analysis of SLC7A11‐bound deubiquitinase eluted from MLO‐Y4 cell lysate. PSM: Peptide Spectrum Match. D) Immunoprecipitation of SLC7A11 or control IgG was performed on MLO‐Y4 cells treated with PBS or DEX (100 µM) for 8 h. E) Immunoprecipitation of PSMD14 or control IgG was performed on MLO‐Y4 cells. F) Immunoprecipitation of the interaction between SLC7A11 and PSMD14 was performed on plasmid‐transfected MLO‐Y4 cells with the indicated antibodies. After transfection was completed, MLO‐Y4 cells were treated with MG132 (10 µM) for 8 h while adding PBS or DEX (100 µM). G) Western blot and quantitative analysis of Flag‐PSMD14 and SLC7A11 protein was performed on MLO‐Y4 cells transfected with indictaed plasmid (n = 5 per group). H) Western blot and quantitative analysis of PSMD14 protein was performed on MLO‐Y4 cells transfected with si‐RNA (50 nM si‐NC or si‐PSMD14 #1‐3 for 24 h) (n = 5 per group). I) Western blot and quantitative analysis of PSMD14 and SLC7A11 proteins were performed on siRNA‐transfected MLO‐Y4 cells (50 nM si‐NC or si‐PSMD14 for 24 h). After transfection was completed, MLO‐Y4 cells were treated with PBS or DEX (100 µM) for 8 h (n = 5 per group). J) Immunoprecipitation of SLC7A11 ubiquitination and its binding to PSMD14 were performed on siRNA‐transfected MLO‐Y4 cells (50 nM si‐NC or si‐PSMD14 for 24 h). After transfection was completed, MLO‐Y4 were treated with MG132 (10 µM) for 8 h while adding PBS or DEX (100 µM). K) Predicted binding complex model of SLC7A11 and PSMD14 (left). The picture showed the hydrogen bonds in the protein interaction region and the corresponding amino acid residues (right). L) Binding energy analysis of the PSMD14‐SLC7A11 complex and the PSMD14‐SLC7A11‐DEX complex based on molecular dynamics simulations. Data are expressed as mean ± SD, with biologically individual data points shown. *p* values were determined by one‐way ANOVA test with Tukey's multiple comparisons (H) and two‐way ANOVA test with Tukey's multiple comparisons (G,I), * *p* <  0.05, ** *p* <  0.01.

As a counterbalance to the UPS, deubiquitinating enzymes (DUBs) collaborate with UPS to maintain cellular protein stability and degradation. To identify SLC7A11‐interacting DUBs, we performed LC‐MS/MS analysis of the purified SLC7A11 complex in MLO‐Y4 cells (**Figure** **4**A). After cross‐referencing with the UbiBrowser repository, we sorted the detected proteins by their coverage, with OTUB1 and PSMD14 deubiquitinases ranking highest, aside from SLC7A11 itself (Figure 4B). In addition to OTUB1 and PSMD14, we identified several other DUBs, including USP10, USP5, COPS5, and USP14, that also bind to SLC7A11 (Table S3, Supporting Information). The coverage and peptide spectrum match (PSM) values in Figure 4C preliminarily suggested OTUB1 and PSMD14 as candidate DUBs interacting with SLC7A11 under DEX treatment.

To confirm this possibility, co‐immunoprecipitation experiments validated that PSMD14 binds to SLC7A11 (Figure 4E), and DEX primarily interferes with the interaction of PSMD14 rather than the known SLC7A11 deubiquitinase OTUB1 (Figure 4D). PSMD14 and SLC7A11 generated by plasmid transfection could interact, and this binding was also inhibited by DEX (Figure 4F,G). Moreover, we used siRNA to knockdown PSMD14 in MLO‐Y4 cells, with the knockdown efficiency shown in Figure 4H. PSMD14 knockdown further amplified DEX‐mediated ubiquitination and expression reduction of SLC7A11 (Figure 4I,J). Notably, PSMD14 expression remained stable and did not decrease after DEX treatment, suggesting that DEX might hinder this interaction to accelerate SLC7A11 degradation.

Subsequently, molecular dynamics (MD) simulations and docking analysis were employed to explore the underlying interaction mechanisms. As shown in Figure S5A,B, Supporting Information, most positions in the PSMD14‐SLC7A11 complex correspond to high sequence values, and the predicted conformation possesses a high confidence score with the sum of ipTM and pTM being 0.856. After analyzing the molecular dynamics trajectory of the steady state (60–100 ns), the protein interfaces of the optimized complexes are complementary in shape, as depicted in Figure S5C, Supporting Information. Collectively, these parameters support that the constructed PSMD14‐SLC7A11 complex is structurally reliable and suitable for further evaluation. Upon introduction of DEX, the drug molecule was primarily located on the SLC7A11 protein of the complex, with interaction forces arising from hydrogen bonds formed by the hydroxyl and carbonyl groups of DEX and the amino acids of SLC7A11, as well as hydrophobic effects (Figure S5H,I, Supporting Information). MD simulation observed that the motion trajectories of DEX and SLC7A11 stabilized after 20 ns, while PSMD14 continued to fluctuate (Figure S5F, Supporting Information). Although the root‐mean‐square fluctuation (RMSF) curves of the binary and ternary complexes have similar trends, the ternary complex exhibited a higher RMSF value (Figure S5G, Supporting Information). The colored 3D structure suggests that DEX enhances the structural flexibility of the complex and reduces its stability (Figure S5D,G, Supporting Information). Additionally, we analyzed the binding sites of the two complexes and simulated their binding free energies to evaluate the impact of DEX on the interaction between PSMD14 and SLC7A11. In the absence of DEX, Lys362, Arg189, and Lys215 of SLC7A11 formed three hydrogen bond connections with Lys310, Glu242, and Glu262 of PSMD14 (Figure 4K). The presence of DEX hindered the formation of these hydrogen bonds, leaving only Leu203 in SLC7A11 connected to Asp256 in PSMD14, and Lys215 to Glu260 and Asp263 (Figure S5E, Supporting Information). As expected, the binding energy profile revealed that DEX significantly amplified the fluctuation range and depressed the baseline of the energy curve by ≈25 kcal mol^−1^ compared to the binary complex (Figure 4L). These data underscore the inhibitory potential of DEX on the assembly of the PSMD14‐SLC7A11 complex.

### 2.5 PSMD14 Stabilizes SLC7A11 by Inhibiting K48‐Linked Polyubiquitination of SLC7A11

**Figure 5:**
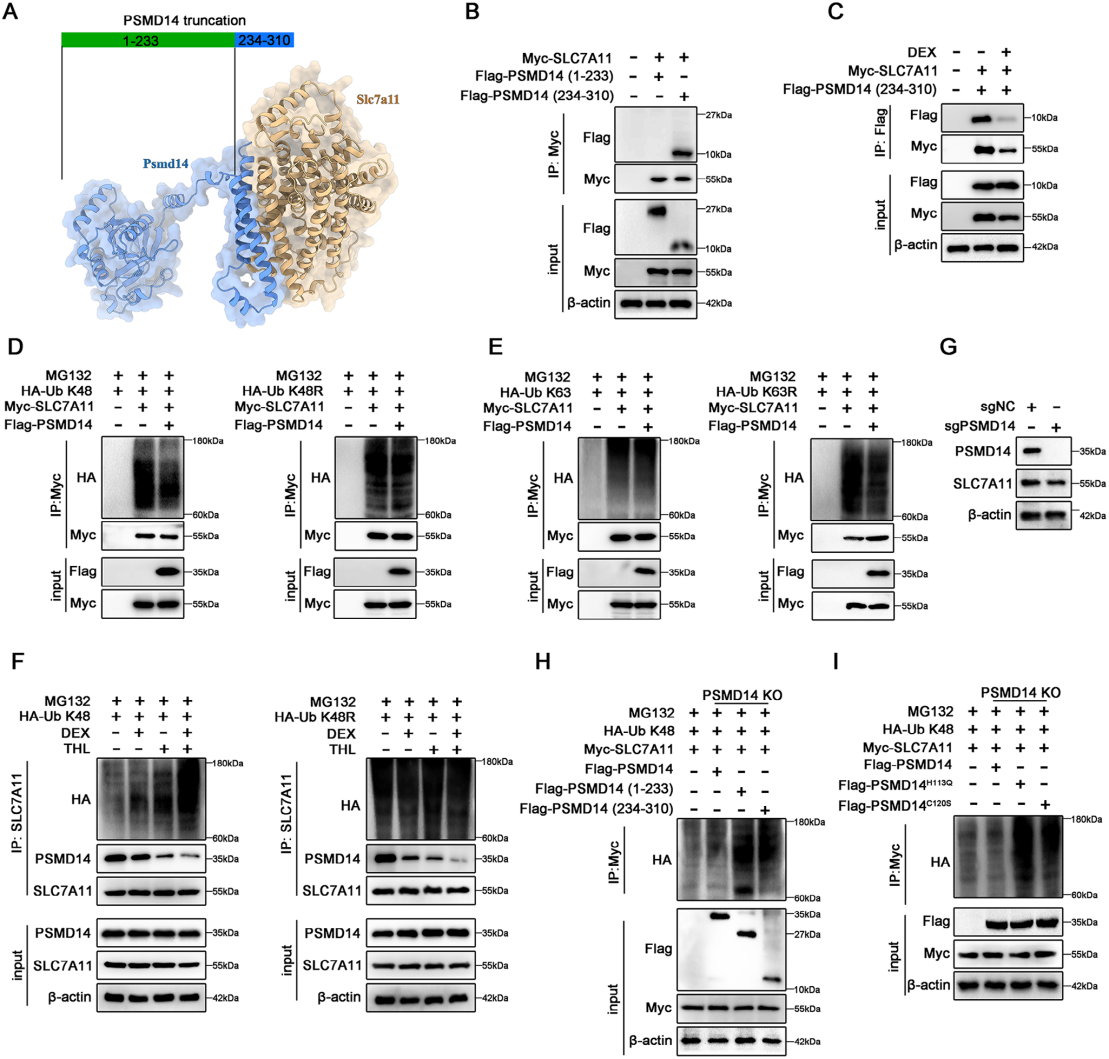
PSMD14 maintains SLC7A11 expression by cleaving K48‐linked polyubiquitin chains from SLC7A11. A) Schematic diagram of the PSMD14‐SLC7A11 protein complex and the PSMD14 mutant plasmids (residues 1–233 and 234–310) used in subsequent immunoprecipitation assays. B) Immunoprecipitation of the interaction between SLC7A11 and each PSMD14 fragment was performed on plasmid‐transfected MLO‐Y4 cells. C) Immunoprecipitation of the interaction between SLC7A11 and PSMD14 (234‐310) fragment was performed on plasmid‐transfected MLO‐Y4 cells. After transfection was completed, MLO‐Y4 cells were treated with PBS or DEX (100 µM) for 8 h. D,E) Immunoprecipitation was performed to identify the type of polyubiquitination of SLC7A11 in MLO‐Y4 cell. MLO‐Y4 cells were cotransfected with Myc‐SLC7A11 plasmid, Flag‐PSMD14 plasmid and HA‐Ub K48/K63 or HA‐Ub K48R/K63R plasmid. After transfection was completed, MLO‐Y4 cells were treated with MG132 (10 µM) for 8 h. F) Immunoprecipitation of SLC7A11 ubiquitination and its binding to PSMD14 was HA‐Ub K48/K48R plasmid‐treated MLO‐Y4 cells. After transfection was completed, MLO‐Y4 cells were treated with MG132 (10 µM) and supplemented with DEX (100 µM) and/or THL (2 µM) for 8 h. G) Stable PSMD14 knockout MLO‐Y4 cells were constructed by CRISPR‐Cas9 strategy. H) Deubiquitination of SLC7A11 requires the intact PSMD14 fragment. PSMD14 knockout MLO‐Y4 cells treated with HA‐Ub K48 plasmid, Myc‐SLC7A11 plasmid, and PSMD14 full‐length or deletion mutant plasmids upon MG132 (10 µM) treatment for 8 h. I) Deubiquitination of SLC7A11 requires the intact PSMD14 fragment. Stable PSMD14 knockout MLO‐Y4 cells were treated with HA‐Ub K48 plasmid, Myc‐SLC7A11 plasmid, and Flag‐PSMD14 or PSMD14 mutants upon MG132 (10 µM) treatment for 8 h. The experiments were repeated three times independently with similar results.

Based on the predicted complex model, we constructed two PSMD14 truncation plasmids (1–233 and 234–310) tagged with Flag to identify the interaction region between PSMD14 and SLC7A11 (**Figure** **5**A). Subsequently, MLO‐Y4 cells were transfected with PSMD14 (1–233)/(234–310)‐Flag plasmids and an SLC7A11‐Myc plasmid. After immunoprecipitation with anti‐Myc antibody, we found that PSMD14 (234–310) bound to SLC7A11, and this interaction was blocked by DEX (Figure 5B,C).

Next, we investigated which type of SLC7A11‐conjugated ubiquitin chains PSMD14 removes. Since K48‐ and K63‐linked chains are the most abundant and functionally characterized polyubiquitin chains reported to date,^[^ ^16^ ^]^ MLO‐Y4 cells were transfected with HA‐tagged K48/K63 ubiquitin chain plasmids (HA‐Ub K48/K63) and HA‐tagged K48R/K63R monomutant ubiquitin chain plasmids (HA‐Ub K48R/K63R). As shown in Figure 5D,E, PSMD14 could remove K48‐linked ubiquitin chains from SLC7A11 but was ineffective against K63‐linked ubiquitin chains. Moreover, K48R downregulated PSMD14‐mediated deubiquitination, while K63R did not affect the enzymatic activity of PSMD14, supporting that PSMD14 specifically clears K48 polyubiquitin chains on SLC7A11. Additionally, we applied Thiolutin (THL, a PSMD14 inhibitor) and DEX to evaluate the effect of PSMD14 on SLC7A11 ubiquitination under pathological conditions. Co‐immunoprecipitation analysis demonstrated that pharmacological inhibition of PSMD14 increased the K48‐linked ubiquitination level of SLC7A11 regardless of DEX presence (Figure 5F). However, when MLO‐Y4 cells were transfected with HA‐Ub‐K48R, the regulatory ability of DEX and THL on SLC7A11 ubiquitination was largely eliminated (Figure 5F), suggesting that PSMD14 primarily interferes with K48‐linked ubiquitin chains on SLC7A11.

Considering that PSMD14 contains multiple domains, we generated a PSMD14 knockout MLO‐Y4 cell line using a CRISPR‐Cas9 strategy to further explore the effect of PSMD14 fragments on SLC7A11 ubiquitination (Figure 5G). Ubiquitin immunoprecipitation assays with fragmented PSMD14 plasmids demonstrated that intact PSMD14 was required for complete deubiquitination of SLC7A11 (Figure 5H). The MPN domain in the N‐terminal segment is known to perform catalytic functions, with histidine at residue 113 and cysteine at residue 120 ensuring enzyme activity.^[^ ^17^ ^]^ Given that PSMD14 (234–310) binds to SLC7A11, we constructed PSMD14 point mutations (PSMD14^H113Q^ and PSMD14^C120S^). As shown in Figure 5I, only wild‐type PSMD14 could clear K48 ubiquitin chains from SLC7A11, whereas point mutants were unable to do so. These data reveal that PSMD14 inhibits K48‐linked polyubiquitination of SLC7A11.

### 2.6 PSMD14 Intervenes in Glucocorticoid‐Induced Ferroptosis and Bone Loss

Although PSMD14 contributes to SLC7A11 stabilization, its role in downstream pathological events remains to be verified. To this end, genetic and pharmacological approaches were employed to interfere with PSMD14 activity in MLO‐Y4 cells, and the ferroptosis process was evaluated using CCK8, western blot, MDA, and C11‐BODIPY assays. Knockdown of PSMD14 by siRNA or inhibition of its enzymatic activity by THL exacerbated DEX‐mediated cell death, loss of SLC7A11 and GPX4 proteins, accumulation of MDA, and lipid peroxides. Conversely, overexpression of SLC7A11 reversed these effects (Figure S6, Supporting Information). These findings confirm that SLC7A11 stabilization is essential for PSMD14‐mediated ferroptosis suppression.

In vivo, intraperitoneal injection of THL further aggravated bone loss, increased lacunar density, trabecular shrinkage, and bone MDA content in GIOP mice (Figure S7, Supporting Information). Building on the bone‐targeting recombinant adeno‐associated virus serotype 9 (rAAV9) developed by Professor Shim,^[^ ^18^ ^]^ we synthesized rAAV9 to overexpress PSMD14 specifically in mouse osteoblast lineage cells, including endosteal osteoblasts and osteocytes. In this construct, the PSMD14 gene sequence was inserted intronically between the CMV enhancer and the EGFP reporter gene (Figure S8A, Supporting Information), allowing for the visualization of successfully transduced cells. As expected, 2 months after intravenous injection of rAAV9.DSS‐Nter‐EGFP into mice, robust expression of EGFP protein was detected in osteocytes within the cortex (Figure S8B, Supporting Information). Moreover, rAAV9.DSS‐Nter‐PSMD14 treatment resulted in ≈1.5‐fold upregulation of *Psmd14* mRNA levels in the tibia compared to rAAV9.DSS‐Nter‐EGFP‐treated mice (Figure S8C, Supporting Information). This corresponded to increased PSMD14 expression in rAAV9.DSS‐Nter‐PSMD14‐treated femurs under DEX exposure, as evidenced by higher SLC7A11 levels and reduced osteocyte lacunae and MDA content (Figure S8D–H, Supporting Information). Micro‐CT analysis revealed that rAAV9.DSS‐Nter‐PSMD14‐treated GIOP mice exhibited increased femoral bone mass, with significant improvements in BV/TV, Tb.Th, Tb.N, and BMD, and a decrease in Tb.Sp compared to rAAV9.DSS‐Nter‐EGFP‐treated GIOP mice (Figure S8F, Supporting Information). Collectively, these results suggest that targeting PSMD14 in osteocytes is beneficial in combating ferroptosis and bone loss.

### 2.7 Screening and Identification of PSMD14 Agonist to Counteract DEX‐Caused Ferroptosis and Osteoporosis

**Figure 6:**
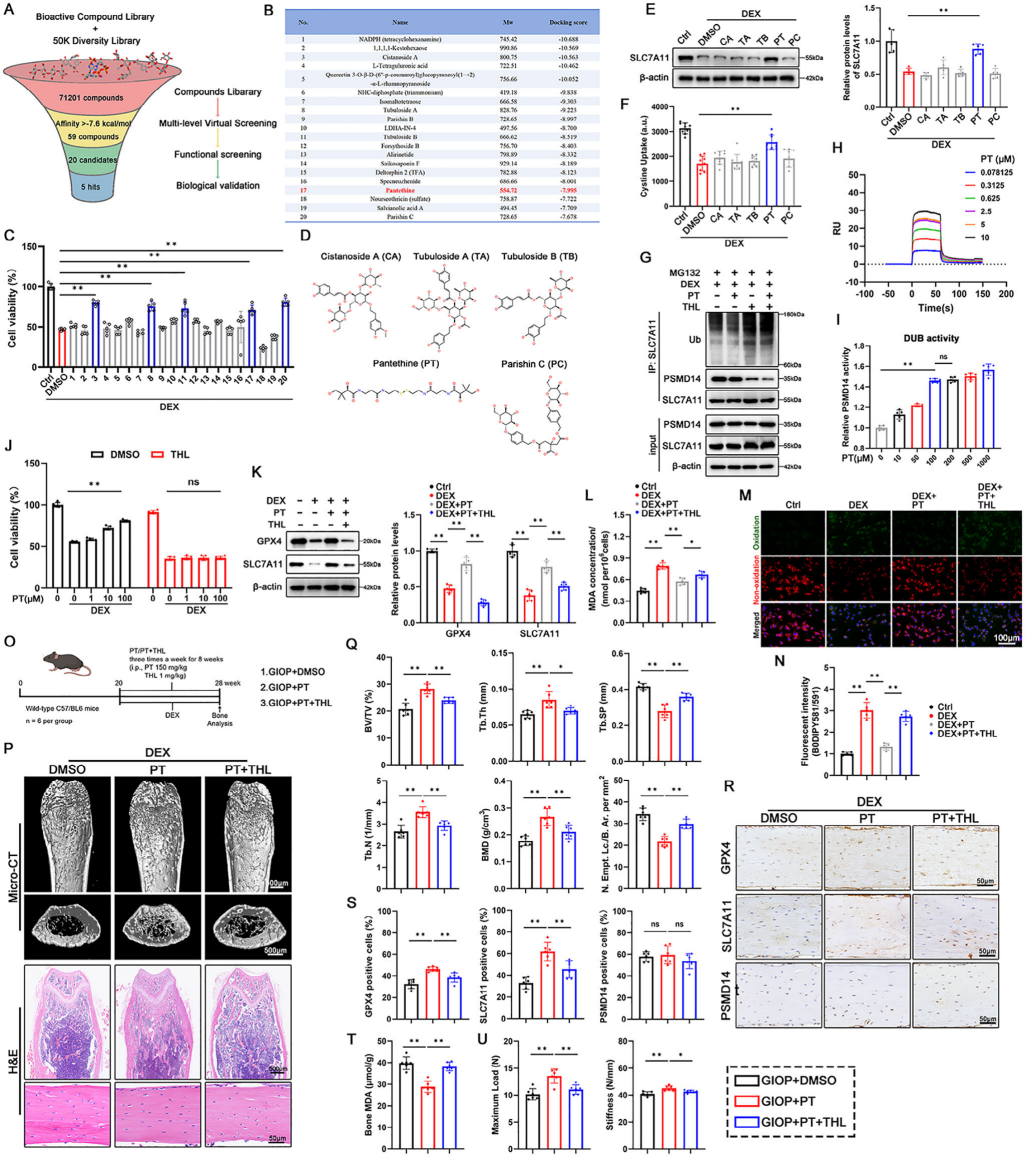
Activation of PSMD14 with Pantethine suppresses osteocyte ferroptosis and bone loss. A) Schematic diagram of screening and identification of PSMD14 agonist. B) Docking scores of the top 20 candidates based on virtual screening. C) CCK‐8 assay was performed on MLO‐Y4 cells treated with different 20 candidates (10 µM) and DEX for 48 h (n = 5 per group). D) Chemical structures of five drug candidates (CA, TA, TB, PT and PC). E) Western blot and quantitative analysis of GPX4 protein were performed on MLO‐Y4 cells treated with DEX (100 µM) supplemented with or wihtout candidates (CA, TA, TB, PT or PC 10 µM) for 48 h. F) Cystine uptake assay was performed on MLO‐Y4 cells treated with DEX (100 µM) supplemented with or wihtout candidates (CA, TA, TB, PT or PC 10 µM) for 48 h (n = 9 per group). G) Immunoprecipitation of SLC7A11 ubiquitination and its binding to PSMD14 were performed on MG132 and DEX‐exposed MLO‐Y4 cells (MG132 10µM and DEX 100 µM). MG132 and DEX‐expousred MLO‐Y4 cells were treated with PT (10 µM) and/or THL (2 µM) for 8 h. H) Binding affinity of PT with recombinant PSMD14 was determined using an SPR assay (K_D_ = 5.14 µM). I) Recombinant PSMD14 were incubated with PT, followed by the measurement of the absorbance at OD 445 nm to detect PSMD14 activity using Ubiquitin‐AMC assay (n = 5 per group). J) CCK‐8 assay was performed on DEX‐exposed MLO‐Y4 cells treated with PT (0–100 µM) and/or THL (2 µM) for 48 h (n = 5 per group). K–N) Western blot and quantitative analysis of GPX4 protein (K), MDA concentration detection (L), C11‐BODIPY 581/591 staining (M) and quantitative analysis (N) were performed on DEX‐exposed MLO‐Y4 cells treated with PT (100 µM) and/or THL (2 µM) for 48 h (n = 5 per group). O) Schematic showing the experimental protocol for 8‐weeks of PT / PT + THL injections in GIOP mice. P) Micro‐CT 3D restruction and H&E staining of the distal femur of mice in each group. The processing details of each group are shown in (O). Q) Distal femur BV/TV, Tb.Th, Tb.Sp, Tb.N, and BMD of mice in each group were measured by micro‐CT (n = 6 per group). Quantitative analysis of the empty lacunae in cortical bone (Number of empty lacunae with respect to bone area, N. Empt. Lc./B. Ar. per mm^2^) based on H&E staining (n = 6 per group). R) GPX4, SLC7A11 and PSMD14 IHC staining of the distal femur of mice in each group. The processing details of each group are shown in (O). S) Quantification of GPX4, SLC7A11 and PSMD14‐positive osteocytes in mouse cortical femurs based on IHC staining (n = 6 per group). T) MDA content in tibia tissue of mice in each group (n = 6 per group). U) Maximum load and Maximum deflection of femoral cortical bone evaluated by the three‐point bending test (n = 6 per group). Data are expressed as mean ± SD, with biologically individual data points shown. *p* values were determined by one‐way ANOVA test with Tukey's multiple comparisons (C,E,F,H,I,L,N,Q,S–U) and two‐way ANOVA test with Tukey's multiple comparisons (J,K), ns, *p* > 0.05, * *p* <  0.05, ** *p* <  0.01.

To preclinically prove the concept mentioned above, we aimed to identify small molecule agonists of PSMD14. Through multi‐level virtual screening of 71 201 small‐molecule drugs from the MedChemExpress Library, we identified chemical compounds capable of binding to the functional pocket of PSMD14. These compounds were scored and then subjected to biological validation (**Figure** **6**A). Sequential SP and XP docking analyses led us to select and purchase 20 candidate compounds with affinities above 7.6 kcal mol^−1^ for subsequent experimental verification (Figure 6B; Table S4, Supporting Information). CCK8 assays revealed that five of these compounds rescued MLO‐Y4 cell viability after DEX treatment (Figure 6C). Their chemical structures and molecular docking are shown in Figure 6D, Figure S9, Supporting Information. Among these five compounds, only Pantethine (PT) significantly stabilized SLC7A11 and accelerated cystine uptake in MLO‐Y4 cells upon DEX stimulation (Figure 6E,F). Five amino acid residues (Pro23, Glu138, Ile163, Ala165, and Gln131) in the cavity center were projected to form hydrogen bonds with PT (Figure S9F, Supporting Information). Co‐immunoprecipitation assays showed that PT decreased the ubiquitination level of SLC7A11, an effect negated by the addition of THL (Figure 6G). Surface plasmon resonance (SPR) assays further confirmed that PT directly bound to recombinant PSMD14 protein with a KD value of 5.14 µM (Figure 6H). Moreover, incubating recombinant PSMD14 protein with PT significantly enhanced PSMD14's DUB activity in a dose‐dependent manner, as measured by the Ubiquitin‐AMC assay (Figure 6I). These data demonstrate that PT, an active Vitamin B5 derivative clinically approved for dyslipidemia,^[^ ^19^ ^]^ stabilizes SLC7A11 by directly binding to PSMD14 and enhancing its deubiquitinating activity in MLO‐Y4 cells.

We further explored the therapeutic potential of PT in vitro and in vivo models and the underlying role of PSMD14. PT treatment significantly inhibited DEX‐mediated lipid peroxide accumulation and loss of anti‐ferroptotic proteins (GPX4 and SLC7A11), as well as prolonged the survival of MLO‐Y4 cells (Figure 6J–N). However, these protective effects of PT were affected by THL. To investigate the function of PT in GIOP mice, PT was injected intraperitoneally three times a week at a dose of 150 mg kg^−1^ for 8 weeks (Figure 6O). The severity of osteoporosis was evaluated by micro‐CT, H&E staining, and three‐point bending test. The results showed that PT treatment increased BMD, the number and thickness of trabecular bone, cortical bone strength, and reduced osteocyte lacunae formation (Figure 6P,Q,U). IHC staining of femurs from PT‐treated mice indicated a dramatic increase in the expression of SLC7A11 and GPX4 compared with GIOP mice, with no effect on PSMD14 expression (Figure 6R,S). Similarly, DEX‐induced MDA levels declined after PT treatment (Figure 6T). However, inhibiting PSMD14 activity with THL directly diminished the PT‐induced anti‐osteoporotic and anti‐ferroptotic effects (Figure 6P–U). Furthermore, we explored the effects of intraperitoneal injection of PT or THL alone on bone quality under physiological conditions. Micro‐CT quantification and H&E staining of femurs after 8 weeks of administration showed that PT or THL alone did not change bone mass or osteocytic morphology (Figure S10A–D, Supporting Information). Notably, H&E staining of the heart, liver, spleen, lung, and kidney indicated that long‐term use of PT did not cause structural abnormalities in major organs (Figure S10E, Supporting Information). In conclusion, PT suppresses GIOP progression and osteocyte ferroptosis by activating PSMD14, which is expected to serve as a candidate pharmaceutical for GIOP therapy.

## 3 Discussion

**Figure 7:**
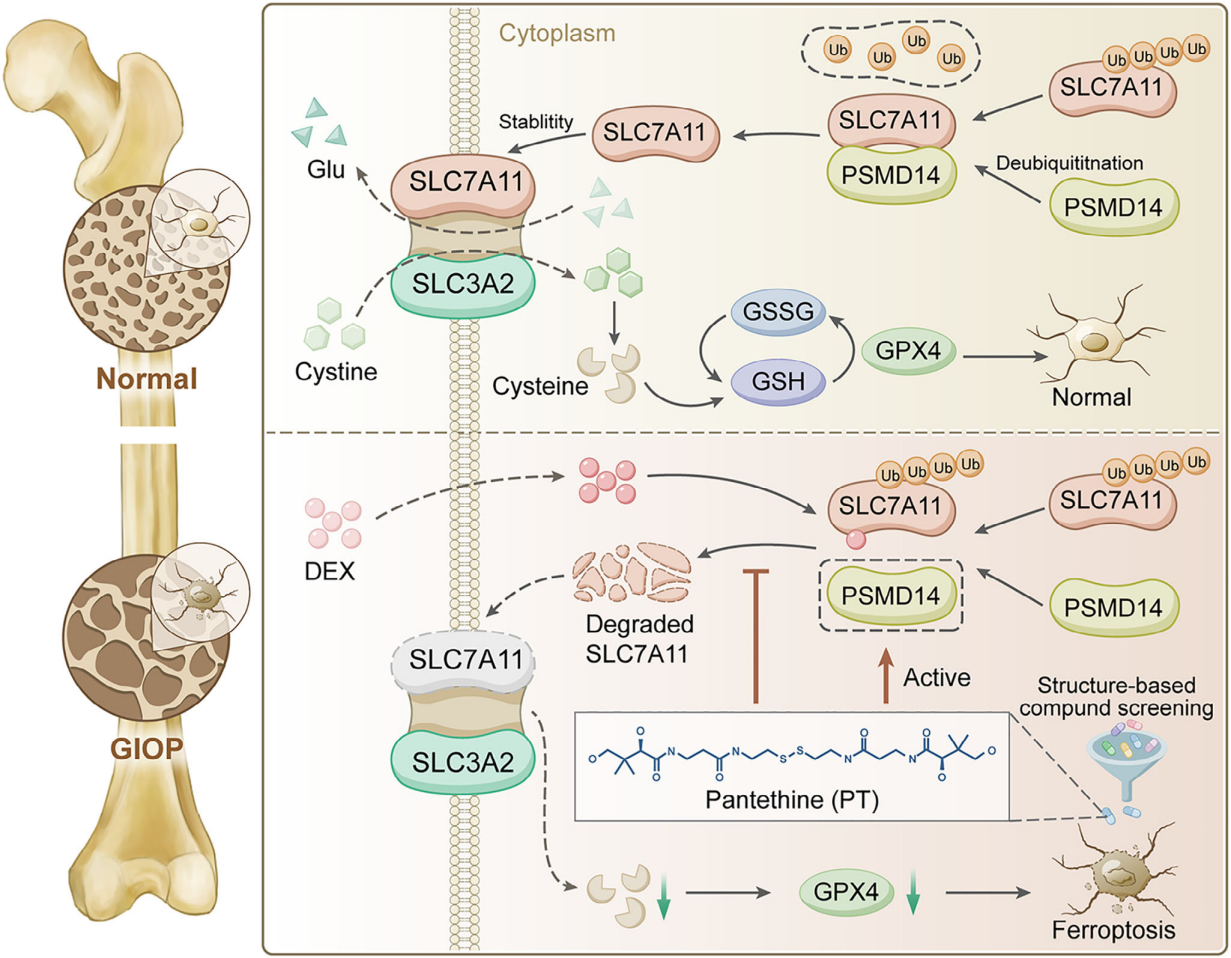
Schematic diagram illustrating the mechanism of GC‐mediated osteocyte ferroptosis. Top: Under physiological conditions, PSMD14 binds to and deubiquitinates SLC7A11 to stabilize SLC7A11 expression and the cystine uptake capacity of osteocytes, thereby ensuring the GSH content and GPX4 activity in osteocytes to maintain cellular function and vitality. Down: During DEX exposure, SLC7A11 is degraded due to limited binding with PSMD14, leading to insufficient cystine in osteocytes and triggering ferroptosis. Combined with virtual screening, we identified PT as a PSMD14 agonist that stabilizes SLC7A11 expression against DEX‐mediated ferroptosis.

Unlike other forms of osteoporosis, GIOP is characterized by more severe bone microarchitectural destruction, which is manifested by increased cortical porosity and bone fragility.^[^ ^3^, ^20^ ^]^ Consistent with these observations, GIOP patients often exhibit higher bone density at the time of fracture compared to those with senile or postmenopausal osteoporosis.^[^ ^21^, ^22^, ^23^ ^]^ The survival and function of osteocytes within bone lacunae under the GC microenvironment have garnered significant attention. In this study, we observed osteocytes with distinct ferroptosis phenotypes, including lipid peroxide accumulation and dysregulation of ferroptosis‐related genes, in GIOP for the first time. Mechanistically, cystine/glutathione metabolic disturbance is crucial for osteocytic ferroptosis, resulting from the restricted binding of PSMD14 to SLC7A11 by GC. Importantly, both genetic and pharmacological targeting of ferroptosis or PSMD14 can reverse GC‐mediated osteocyte death and bone loss by stabilizing SLC7A11 (**Figure** **7**).

Bone loss and fracture risk rapidly intensify in the initial phase of GC therapy.^[^ ^1^, ^6^ ^]^ Our results, along with those of others, suggest that GCs lead to the enlargement of osteocyte lacunae, which microscopically undermines bone material strength. This phenomenon potentially explains the increased fracture risk in GIOP despite similar bone density levels. However, the mechanisms by which GCs cause these changes have not been fully described. Accordingly, by focusing on the distinctive osteocytes within cortical bone, our proteomic analysis revealed that GCs induce aberrations in pathways associated with ferroptosis, lipid peroxidation, and glutamine metabolism within these cells. Similar evidence has been found in ONFH caused by GCs. For example, differential enrichment of ferroptotic genes was found in the serum of ONFH patients (GSE123568), and methylprednisolone stimulation destroyed intracellular mitochondria in bone marrow stromal cells (BMSCs) to accelerate lipid peroxidation damage.^[^ ^11^, ^13^ ^]^ However, these findings may not be sufficient to conclude that ferroptosis is involved in the pathogenic process of GC‐induced bone destruction, as the aforementioned mitochondrial damage and oxidative gene differences lack specificity. The International Cell Death Nomenclature Committee defines ferroptosis as GPX4‐regulated cell death initiated by intracellular oxidative disturbances, which can be inhibited by both iron chelators and lipophilic antioxidants.^[^ ^24^ ^]^ Under physiological conditions, GPX4 knockout does not directly destroy osteocytes or alter bone microstructure. In GIOP modeling mice, GPX4‐deficient osteocytes experienced severe ferroptosis, and the mice's bone mass and bone microarchitecture were further deteriorated, indicating that osteocyte ferroptosis promotes GIOP development. Meanwhile, the classic inhibitors Fer‐1 and DFO reversed DEX‐mediated lipid peroxide accumulation, osteocyte ferroptosis, and bone destruction, which was also beneficial in mice and MLO‐Y4 cells with GPX4 knockout. Interestingly, it has been speculated that ferroptosis and other forms of cell death may crosstalk or even transform into each other. Osteocyte apoptosis occurs in GC‐treated human and mouse bones.^[^ ^25^, ^26^ ^]^ Given the potential involvement of GPX4 in apoptosis and necroptosis, further studies are warranted to uncover the subtle interactions between different types of osteocyte death in GIOP.

While our study primarily focused on osteocyte ferroptosis as a central mechanism, emerging evidence suggests that GCs exert multifaceted effects on BMSCs, osteoblasts, and osteoclasts. Notably, ferroptosis features—mitochondrial damage, lipid peroxidation, and reduced antioxidant capacity—were observed in GC‐treated BMSCs and preosteoblasts, impairing osteogenic differentiation. For instance, DEX downregulates SIRT3 to induce mitophagy and fission in MC3T3‐E1 pre‐osteoblasts, led to mitochondrial dysfunction.^[^ ^27^ ^]^ Transcription factors such as STAT3 and Nrf2 mediate the expression of antioxidant proteins (e.g., GPX4 and SOD2), which underlie DEX‐induced ferroptosis in BMSCs and osteoblasts.^[^ ^28^, ^29^ ^]^ This ROS crisis is compounded by GDF15‐mediated redox imbalance and PDK4‐driven glycolytic reprogramming, collectively priming BMSCs for ferroptosis.^[^ ^13^, ^30^ ^]^ Even in osteoclasts, RANKL stimulation elevates intracellular ferrous iron and ROS—hallmarks of ferroptosis—while iron chelators like DFO inhibit differentiation by blocking mitochondrial electron transport.^[^ ^31^, ^32^, ^33^ ^]^ But direct evidence linking GCs to osteoclast's ferroptosis remains elusive. These findings collectively suggest that GCs exploit ferroptosis‐prone vulnerabilities in diverse bone cells, though the molecular triggers may vary by cell lineage.

Other GC‐related ferroptosis studies, including those on allergic airway inflammation and ONFH, have attributed the ferroptotic upstream to several functional events such as ROS accumulation and mitochondrial damage.^[^ ^13^, ^34^ ^]^ However, the precise molecular mechanism has not yet been fully elucidated. Ferroptosis‐inducing agents (FINs) are categorized into type I FINs, which involve GSH depletion (e.g., Erastin), and type II FINs, which trigger ferroptosis by inhibiting GPX4 (e.g., RSL3).^[^ ^15^, ^35^, ^36^ ^]^ Our findings indicate that DEX downregulates SLC7A11 expression to inhibit cystine uptake in MLO‐Y4 cells, thereby blocking endogenous glutathione synthesis. Professor Linkermann reported that DEX halved the GSH content in HT1080 cells by accelerating the degradation of cysteinylglycine, but DEX only sensitized Erastin‐induced ferroptosis rather than directly triggering ferroptosis.^[^ ^37^ ^]^ In their study, no cell death was detected by 7‐AAD/annexin V flow cytometry even when HT1080 cells were treated with 100 µM DEX alone for 50 h. This discrepancy may be explained by differences in death detection methods and cell tolerance. The CCK8 assay used in our study reflects the number of viable cells currently in the well plate, which may be slightly affected by the proliferation of MLO‐Y4 cells during DEX treatment. Nevertheless, our application of Fer‐1 and DFO, as well as lipid peroxidation and GPX4 detection, further confirmed the ferroptosis potential of DEX in MLO‐Y4 cells. On the other hand, the MLO‐Y4 cell line, which originates from osteoblast‐like cells in the transgenic mouse skeleton, might be less resistant to DEX than the highly proliferative and invasive HT1080 cells (derived from human fibrosarcoma).^[^ ^38^ ^]^ Notably, these contradictions do not hinder the consistent conclusion that intracellular GSH content is a key initiator of DEX‐regulated ferroptosis.

Based on the above research, we investigated how DEX depletes GSH in MLO‐Y4 cells. Metabolomics analysis revealed that the restriction of glutathione biosynthesis is characterized by the decline of intermediates, including cystine, cysteine, and γ‐glutamylcysteine. Through a series of in vitro and in vivo experiments, we demonstrated that DEX reduced the protein expression of SLC7A11 while the mRNA level remained basically unchanged. In addition to transcriptional regulation, emerging evidence indicated potential involvement of post‐translational modifications, including O‐GlcNAcylation, palmitoylation and ubiquitination, in modulating SLC7A11 function.^[^ ^39^, ^40^, ^41^ ^]^ By applying CHX and MG132 in MLO‐Y4 cells, we confirmed that DEX destabilizes SLC7A11 by promoting ubiquitination and degradation. Differently, Peng et al.^[^ ^42^ ^]^ suggested that DEX inhibits SLC7A11 expression in MLO‐Y4 cells by upregulating p53, a transcription factor that interferes with the SLC7A11 promoter in the nucleus. However, they lack direct supporting evidence, for example, the mRNA level of SLC7A11 has not been detected. Since ovarian tumor deubiquitinase B1 (OTUB1) was identified as a stabilizer of SLC7A11, related E3 ligases and deubiquitinating enzymes have been discovered, such as SOCS2, TRIM3, and ZRANB1.^[^ ^41^, ^43^, ^44^, ^45^ ^]^ Although OTUB1 was the most abundant deubiquitinase bound by SLC7A11 according to our LC‐MS analysis, this interaction did not appear to be interfered by DEX. Instead, the second‐ranked PSMD14, a member of the JAMM metalloprotease family, is the target of DEX‐promoted SLC7A11 degradation. Inhibition of PSMD14 by siRNA or THL further aggravated SLC7A11 degradation and ferroptosis occurrence in DEX‐treated MLO‐Y4 cells. Mice overexpressing PSMD14 exhibited greater bone mass and denser cortical bone, suggesting its potential for GIOP.

The subsequent inquiry pertains to the underlying mechanism by which DEX diminishes the formation of the PSMD14‐SLC7A11 complex. Classically, DEX interacts with the glucocorticoid receptor (GR) to form a dimerized complex, which then translocates to the nucleus and regulates downstream gene transcription.^[^ ^46^ ^]^ However, no significant difference in PSMD14 expression was detected after DEX treatment. Given its lipid solubility, we speculated that DEX may act as a molecular clamp to block the binding of PSMD14 to SLC7A11. Notably, we predicted and essentially determined the binding mode of PSMD14 and SLC7A11 for the first time, thereby expanding our understanding of PSMD14. Functional studies and structural analysis (UniProt) of the protein confirmed that the JAMM motif contained in the MPN domain (approximately residues 31–166) and the C120 and H113 sites are the core components maintaining the deubiquitinating activity of PSMD14.^[^ ^47^ ^]^ Aberrant mutations at the C120 or H113 sites of PSMD14 failed to deubiquitinate SLC7A11, which is consistent with previous studies.^[^ ^48^, ^49^ ^]^ The estrogen receptor α interacts with the residues 1–172 region of PSMD14 (including the MPN domain) to remove polyubiquitin chains.^[^ ^48^ ^]^ Interestingly, Liu et al.^[^ ^50^ ^]^ found that neither the JAMM motif nor the MPN domain deletion interfered with PSMD14 binding to substrate proteins. Similarly, we found that PSMD14 mutants lacking the MPN domain (PSMD14 (234–310) plasmid) were still able to bind SLC7A11, but this interaction appeared to be weakened after DEX treatment. Due to the complexity of the PSMD14‐SLC7A11 binary complex, it is challenging to elucidate the anchor point of the DEX‐blocking complex based solely on molecular biology experiments. Molecular dynamics (MD) simulation appears to be a viable approach. DEX was introduced into the binary complex via docking technology, and the molecular motion trajectory was simulated to analyze the binding energy and mode of the PSMD14 and SLC7A11 proteins. The presence of DEX affects the hydrogen bond structure of the protein binding interface, causing the motion trajectory of the PSMD14 and SLC7A11 proteins to oscillate and the binding energy to decrease. Of course, since the crystal structures of PSMD14 and SLC7A11 have not been decoded, the protein complex model constructed based on AlphaFold software may be distorted.

Genetic overexpression of PSMD14 protects GIOP mice from ferroptosis and bone loss, but pharmacological regulation appears more promising. Several PSMD14 inhibitors have been applied in tumor research, yet the development of PSMD14 agonists remains elusive.^[^ ^51^ ^]^ In this work, we combined virtual screening and functional experiments to identify the small‐molecule agonist Pantethine, a vitamin B5 precursor that targets PSMD14 to stabilize SLC7A11, and demonstrated its anti‐ferroptosis and anti‐osteoporosis properties. Among the B vitamins, only B2, B6, and B12 have been proven to be related to osteoporosis.^[^ ^52^ ^]^ Perhaps due to the limitations of vitamin B5 detection technology, its role in osteoporosis has not been extensively reported. The synthesis of coenzyme A, which is essential for multiple metabolic reactions including the tricarboxylic acid cycle and fatty acid metabolism, begins with vitamin B5.^[^ ^53^ ^]^ PT, an approved lipid‐lowering agent, has shown potential in Phase III clinical trials for the treatment of pantothenate kinase‐associated neurodegeneration.^[^ ^54^, ^55^ ^]^ Our work is the first to demonstrate the targeted activation of PSMD14 by PT and to elucidate the relationship between PT and ferroptosis, thereby expanding the pharmacological mechanisms of PT and providing compelling evidence for its antagonistic effects against GIOP.

Current therapeutic strategies for GIOP, including bisphosphonates, teriparatide, and denosumab, primarily target osteoclast‐mediated resorption or osteoblast anabolism. However, these approaches fail to address GC‐induced osteocyte death and lacunar‐canalicular network disruption, leaving residual fracture risks despite stabilized BMD. Moreover, long‐term use of bisphosphonates carries risks of osteonecrosis of the jaw, while teriparatide requires invasive administration and raises concerns about osteosarcoma.^[^ ^56^ ^]^ Our study offers a paradigm shift by targeting ferroptosis—a novel mechanism underlying osteocyte dysfunction. By stabilizing SLC7A11 through PSMD14 activation, our approach directly mitigates osteocytic ferroptosis and preserves bone microstructure integrity. Pantethine, a repurposed vitamin B5 derivative with established safety profiles, presents a clinically translatable solution.

In summary, we found that osteocytes undergo ferroptosis upon GC exposure, which aggravates GC‐mediated bone loss. Mechanistically, GC promotes SLC7A11 ubiquitination and degradation by limiting PSMD14 binding. Building on this mechanism, we identified PT as a PSMD14 activator that mitigates GC‐induced ferroptosis and bone deterioration, establishing its potential as a therapeutic candidate for GIOP.

## 4 Experimental Section

### Patient and Specimens

The human studies were approved by the Ethics Committee of the Second Affiliated Hospital of Wenzhou Medical University (Approval Number: 2024‐K‐316‐01). Written informed consent was obtained from all participants before the procedure. Human bone samples were collected from 16 male patients who were undergoing hip replacement surgery due to femoral neck fractures. All patients had undergone bone density and femoral CT scans prior to the surgery. Femoral neck samples with normal bone density were classified as the Normal group (ages 55–73). Samples from patients with osteoporosis who had been taking GC at a dose of at least 7.5 mg day^−1^ for more than 6 months were designated as the GIOP group (ages 45–66). Each bone sample was divided into two parts: one part was decalcified for histological staining, while the other was reserved for the assessment of MDA content.

### Mouse Strains and Treatment

The experimental protocols were approved by the Animal Care and Use Committee of Wenzhou Medical University (Approval number: wydw2024‐0519). C57BL/6 mice were provided by the Animal Center of Wenzhou Medical University, while GPX4^fl/fl^ (strain No. T050827) and Dmp1‐iCre (strain No. T004830) mice were purchased from GemPharmatech (Nanjing, China). To generate GPX4 conditional knockout (Dmp1‐iCre; GPX4^fl/fl^) mice, GPX4^fl/fl^ mice were crossed with Dmp1‐iCre mice. Genotyping was performed via Real‐time quantitative PCR (qPCR) analysis on DNA extracted from mouse tail samples, with the primer sequences detailed in Table S5, Supporting Information. All mice were housed in temperature‐controlled microisolator cages at the Experimental Animal Center of Wenzhou Medical University, under a 12‐h light/dark cycle with ad libitum access to water and food. To minimize estrogen interference, only male mice were selected for subsequent experiments.

The GIOP mice model was established as previously described.^[^ ^57^ ^]^ Specifically, 20‐week‐old wild‐type C57BL/6 and transgenic mice were subcutaneously injected with either 2.5 mg kg^−1^ DEX (MedChemExpress, Shanghai, China) or PBS as a control, three times weekly for 8 weeks. Following the treatment period, the mice were humanely euthanized. Their femurs were collected for micro‐CT scanning and histological staining, and the tibiae were harvested for MDA content determination.

### Cell Culture and Treatment

MLO‐Y4 cells were cultured in α‐MEM (Gibco, Shanghai, China), which was supplemented with 1% penicillin/streptomycin (Gibco) and 10% fetal bovine serum (Gibco). The cells were then incubated in a 5% CO₂ incubator at 37 °C. To establish an osteocyte ferroptosis model, MLO‐Y4 cells were treated with various concentrations of DEX. Cell viability was subsequently assessed using the CCK‐8 assay (MedChemExpress). To induce ferroptosis, MLO‐Y4 cells were treated with 100 µM DEX for 48 h. For experiments related to ubiquitination, the duration of DEX treatment was reduced to 8 h.

### Construction of PSMD14‐Knockout Cell Lines

To generate a stable PSMD14 knockout cell line in MLO‐Y4 cells, a dual‐vector CRISPR‐Cas9 system was utilized. Initially, the cells were transduced with a viral supernatant containing the lentiCas9‐Blast vector, together with the required packaging plasmids (pMDLg/pRRE, pRSV‐Rev, and pMD2.G). Post‐transduction, cells were selected using blasticidin (5 µg mL^−1^, MeilunBio) to isolate those that had been successfully transduced. Once individual cells had proliferated into colonies, Western blotting was employed to confirm Cas9 expression, thereby ensuring efficient integration and expression of the Cas9 protein. Subsequently, guide RNAs (sgRNAs) targeting the PSMD14 gene were cloned into the lentiGuide‐Puro vector. The sgRNA sequences (sgRNA‐1: 5’‐ATATGCTGAAACAAACAGGA‐3’; sgRNA‐2: 5’‐TGCCACAGCTCTCTCCGACA‐3’) were designed using the Optimized CRISPR Design Tool (Zhang Lab, MIT) to maximize targeting specificity. Cas9‐expressing MLO‐Y4 cells were then transduced with a viral supernatant containing the lentiGuide‐Puro vector and packaging plasmids. Following this transduction, cells were selected using puromycin (1 µg mL^−1^; MeilunBio) to enrich for cells carrying the knockout construct. Finally, the efficacy of the PSMD14 knockout was assessed at the protein level in individual cells, confirming the successful establishment of a stable PSMD14 knockout cell line.

### Transfection of Plasmids and siRNAs

A suite of siRNAs targeting PSMD14 and GPX4, as well as plasmids encoding full‐length Flag‐tagged PSMD14 and its deletion mutants (Flag‐PSMD14 (1–233), Flag‐PSMD14 (234–310)), and specific mutants (Flag‐PSMD14^H113Q^, Flag‐PSMD14^C120S^), were sourced from Fenghui Biotechnology (Changsha, China). In parallel, plasmids expressing Myc‐tagged SLC7A11 and a series of HA‐tagged ubiquitin variants (HA‐Ub, HA‐K48, HA‐K48R, HA‐K63, and HA‐K63R) were also obtained. These constructs were generated by cloning the corresponding cDNA sequences into the pcDNA 3.1(+) expression vector. Transfections were performed using Lipofectamine 3000 reagent (Invitrogen, CA, USA), following the manufacturer's protocols to ensure optimal transfection efficiency and minimize cellular toxicity. The specific sequences of the siRNAs used in this study are provided in Table S6, Supporting Information.

### Transduction of Adeno‐Associated Viral Vectors In Vitro

Recombinant AAV vectors encoding SLC7A11 or GPX4 were obtained from Fenghui Biotechnology. To establish a baseline for transduction efficiency, a control group was set up using an empty vector. MLO‐Y4 cells were seeded in six‐well plates and incubated overnight to ensure proper adherence. The following day, the cells were transduced with the AAV vectors for 48 h to promote gene expression. To select cells that had successfully incorporated the vectors, puromycin was added to the culture medium at a final concentration of 2 µg mL^−1^. This selection process enriched the population of cells expressing the transduced genes. The efficacy of transduction and expression of the respective transgenes were confirmed by Western blot analysis, which verified the presence of the corresponding proteins.

### MDA Assay

The MDA content in tibia tissue and MLO‐Y4 cells was evaluated using the Lipid Peroxidation MDA Assay Kit (Beyotime, Shanghai, China). Tibia samples were first stripped of bone marrow, then homogenized and centrifuged to obtain a supernatant for subsequent analysis. MLO‐Y4 cells were harvested, counted, and lysed in radioimmunoprecipitation assay (RIPA) buffer (Beyotime), followed by centrifugation to collect the supernatant for further use. The MDA measurement was performed in strict accordance with the manufacturer's protocol.

### Assessment of Lipid Peroxidation

MLO‐Y4 cells were seeded in six‐well plates at optimal densities and incubated overnight to ensure proper cell adherence. The following day, the cells were treated according to the experimental protocol. Subsequently, the cells were incubated with 5 µM BODIPY 581/591 C11 (Invitrogen) at 37 °C for 30 min to assess lipid peroxidation. The fluorescence intensity of the cells, which indicates lipid peroxidation levels, was measured using a confocal scanning microscope (Zeiss, Jena, Germany), known for its high sensitivity as a ratiometric reporter.

### Cystine Uptake

In accordance with the manufacturer's protocol from Dojindo (Kumamoto, Japan), this work assessed the system activity in MLO‐Y4 cells using the Cystine Uptake Assay Kit. Cells were seeded in black 96‐well plates at a density of 10,000 cells per well and allowed to adhere overnight. Following treatment, the cells were washed and incubated with α‐MEM medium (cystine‐free, serum‐free) at 37 °C for 5 min to equilibrate the temperature. Subsequently, the cells were incubated in the same α‐MEM medium supplemented with cystine analogs for 30 min at 37 °C. The cells were then washed with PBS to remove any unreacted analogs. The assay working solution was added, and the plates were incubated for an additional 30 min at 37 °C to develop the colorimetric reaction. The fluorescence intensity of each group (Ex/Em = 490/535 nm) was measured using a fluorescence microplate reader (Thermo Scientific, MA, USA). Wells that did not receive cystine analogs served as the blank control group.

### Glutathione Detection

MLO‐Y4 cells were plated in six‐well plates at optimal densities and permitted to adhere overnight. The cells were then treated with 100 µM DEX for 48 h. Following treatment, the cells were carefully harvested from each well. The levels of total glutathione (GSH) and its oxidized form (GSSG) in the collected supernatants were quantified using the GSSG/GSH Quantification Kit (Dojindo), strictly adhering to the manufacturer's protocol.

### Immunoblot and Immunoprecipitation

After treatment, MLO‐Y4 cells were lysed to extract total protein using RIPA buffer supplemented with protease inhibitors (Roche Basel, Switzerland) to ensure complete protein solubilization. Protein concentrations in the lysates were quantified using a BCA protein assay kit (Beyotime), enabling standardized sample preparation. Equal amounts of protein (30 µg) from each sample were resolved by SDS‐PAGE and electrophoretically transferred onto PVDF membranes. The membranes were blocked with 5% skim milk to prevent non‐specific antibody binding and then incubated with specific primary antibodies (Table S7, Supporting Information) at 4 °C overnight. This extended incubation period enhances antibody binding efficiency. Following incubation with the primary antibodies, the membranes were incubated with corresponding horseradish peroxidase‐conjugated secondary antibodies (Table S7, Supporting Information). The immune complexes were visualized using an enhanced electrochemiluminescence Beyo ECL plus reagent (Beyotime) after extensive washing with 0.05% Tris‐buffered saline/Tween (TBST) to remove unbound secondary antibodies.

For co‐immunoprecipitation, lysates were incubated with specific primary antibodies (Table S7, Supporting Information) at 4 °C overnight. The immune complexes were then captured using Pierce protein A/G magnetic beads (Thermo Scientific), which were subsequently washed to enrich and recover the bound proteins. The immunoprecipitated proteins were resolved by SDS‐PAGE and analyzed by immunoblotting to detect the presence of interacting proteins.

### Protein Stability and Ubiquitination Assay

To determine the half‐life of endogenous SLC7A11 in MLO‐Y4 cells under the influence of DEX, a cycloheximide (CHX, MedChemExpress) chase assay was performed. Cells were initially treated with 100 µM DEX to modulate SLC7A11 expression. Subsequently, 50 µg mL^−1^ CHX was added to inhibit further protein synthesis. Aliquots of cells were harvested at 0, 2, 4, and 8 h post‐CHX treatment to assess SLC7A11 degradation over time. Cell lysates were then subjected to immunoblotting using a specific antibody against SLC7A11 to monitor protein levels.

To evaluate the deubiquitinating activity of PSMD14 on SLC7A11, MLO‐Y4 cells were co‐transfected with si‐PSMD14 or relevant plasmids. After a 24‐h transfection period, cells were treated with 10 µM of the proteasome inhibitor MG132 (MedChemExpress) for 8 h to accumulate ubiquitinated proteins prior to sample collection. The ubiquitination status of SLC7A11 was then assessed using co‐immunoprecipitation.

### RNA Extraction and qPCR Analysis

Total RNA was isolated from tibia and MLO‐Y4 cells using the RNA Isolater Total RNA Extraction Reagent (Vazyme, Nanjing, China), in accordance with the manufacturer's instructions. The quantity and purity of the RNA were evaluated by measuring the absorbance at 260 nm and calculating the 260/280 nm ratio with a NanoDrop spectrophotometer (NanoDrop Technologies, Wilmington, DE, USA). RNA samples with a 260/280 ratio between 1.8 and 2.0 were deemed suitable for further analysis. One microgram of high‐quality RNA from each sample was reverse‐transcribed into cDNA using a cDNA synthesis kit (Takara, Shiga, Japan). Real‐time quantitative PCR (qPCR) was conducted using the SYBR Green q‐PCR Kit (Vazyme) on a CFX‐96 system (Bio‐Rad, CA, USA), following the recommended protocol. Gene expression levels were quantified using the 2^‐ΔΔCt^ method and normalized to the housekeeping gene Actb (β‐actin). Amplification efficiency was assessed using the standard curve method. Primer sequences for all target genes are provided in Table S8, Supporting Information.

### Whole Mount Alcian Blue/Alizarin Red Staining for Mouse Embryos

On postnatal day 0 (P0), mouse embryos were prepared by first removing the skin, viscera, and muscle. Following this, the embryos were fixed in 95% ethanol overnight. After fixation, the embryos were degreased in absolute acetone for a duration of 16 to 24 h. The staining process was then carried out using a solution that contained Alcian Blue and Alizarin Red (Servicebio, Wuhan, China) in 70% ethanol, with the embryos being stained overnight. To remove excess stain, the embryos were washed in 70% ethanol for 30 min, and this washing step was repeated three times. Subsequently, soft tissue digestion was performed using a 1% KOH solution. After digestion, the embryos were cleared in a solution composed of 50% glycerol and 50% (1%) KOH. Finally, the stained embryos were stored in 100% glycerol for imaging purposes.

### Bone‐Targeting rAAV9‐PSMD14 Plasmid Construction

This work commissioned Fenghui Biotechnology to construct a recombinant bone‐targeted rAAV9‐PSMD14 plasmid. Specifically, the bone‐targeting rAAV9 vector (rAAV9.DSS‐Nter) was generated by inserting the codon‐optimized DNA sequence encoding the bone‐targeting peptide motif, DSS (Asp‐Ser‐Ser)_6_, into the AAV9 capsid protein VP2, as previously described.^[^ ^18^ ^]^ The gene sequences of PSMD14 were assembled using Gibson assembly and standard molecular biology techniques. These constructs were subsequently cloned into the intronic region of the pAAV‐CMV‐EGFP plasmid at the restriction enzyme sites for PstI and BglII. The accuracy of the cloned constructs was verified by DNA sequencing, after which they were packaged into the rAAV9.DSS‐Nter vector. The production of rAAV9 was achieved by transiently transfecting HEK293 cells. The vector was purified using CsCl density gradient centrifugation and quantified by droplet digital PCR on a QX200 ddPCR system (Bio‐Rad) with an EGFP primer/probe set, as detailed in previous studies.^[^ ^58^ ^]^

### Micro‐CT Analysis

Micro‐CT scanning and quantitative analysis of femurs were performed using the SkyScan 1276 system (Bruker MicroCT, Kontich, Belgium), equipped with proprietary software. Image acquisition was conducted at a voxel size of 9 microns, using an X‐ray source set at 35 kV and 220 mA. 2D and 3D reconstructions of the acquired images were completed using DataViewer and CTVox software, respectively. For the analysis, the trabecular bone region above the growth plate of the distal femur (200 slices, 1.8 mm) was selected as the region of interest (ROI). This selection focuses on the bone area most susceptible to changes in density and structure. Quantitative measurements of bone mineral density (BMD), trabecular bone volume fraction (BV/TV), trabecular number (Tb.N), trabecular thickness (Tb.Th), and trabecular separation (Tb.Sp) were determined using CTAn software, providing detailed insights into the micro‐architectural properties of the trabecular bone.

### Three‐Point Bending Test

The strength of the cortical bone in mouse femurs was assessed using a three‐point bending system (MTS 858, USA). Freshly harvested femoral cortical bone samples were wrapped in gauze soaked in saline to maintain hydration. Specimens were positioned horizontally on two lower supports with a 6‐mm span, ensuring that the loading probe contacted the anteroposterior midpoint of the diaphysis. A vertical load was applied at a rate of 1 mm min^−1^ until fracture occurred. The maximum load and maximum deflection were subsequently analyzed.

### Histopathological Analysis

After fixation in paraformaldehyde and decalcification with EDTA, the femurs were embedded in paraffin. Serial sections, each 4 µm thick, were prepared using a microtome. Hematoxylin and eosin (H&E) staining was performed according to established protocols to visualize bone lacunae and osteocytes, providing a morphological assessment of the bone tissue.^[^ ^59^ ^]^

For immunohistochemical (IHC) staining, the sections were first deparaffinized to remove paraffin and then underwent antigen retrieval to expose antigenic sites. After blocking non‐specific binding, the sections were incubated with the indicated primary antibodies (Table S7, Supporting Information) targeting proteins of interest. This was followed by the application of secondary antibodies conjugated to a detection system. Semi‐quantitative analysis of the IHC staining was conducted using Image‐Pro Plus software version 6.0 (Media Cybernetics, MD, USA).

### Proteomic Analysis of Mouse Femur

Midshaft femoral cortices were prepared for proteomic analysis as described in previous studies.^[^ ^60^, ^61^ ^]^ Murine femora were meticulously scraped with surgical blades to ensure complete removal of the periosteum and disruption of surface‐adherent cells. This was followed by excision of the epiphyseal ends and lavage of the marrow cavity with PBS until diaphyseal whitening was achieved. Demineralization of the femurs was accomplished by incubating them in 1.2 M HCl at 4 °C overnight. Afterward, the bones were washed and homogenized using a tissue homogenizer in an extraction buffer containing 6 M guanidine hydrochloride, 10 mM Tris‐HCl, and 50 mM EDTA. Protein extraction was performed via ultrafiltration centrifugation, and the protein concentration of each femur was measured. The extracted proteins were subjected to the Filter Aided Sample Preparation (FASP) method for hydrolysis. Enzymatically derived peptides were desalted using a C18 cartridge, lyophilized, and reconstituted with 40 µL of dissolution buffer for quantification. For isotopic labeling, 100 µg of peptides from each sample were processed using the TMT kit (Thermo Scientific). To reduce sample complexity, the labeled peptides were fractionated using an AKTA Purifier 100 (Thermo Scientific). The fractionated samples were then loaded onto a nano‐flow HPLC system, Easy nLC (Thermo Scientific), and analyzed using a Q‐Exactive mass spectrometer (Thermo Scientific). HPLC separation was conducted with mobile phase A consisting of 0.1% formic acid and mobile phase B, a 0.4% formic acid solution in 84% acetonitrile. A gradient elution program was applied (0–35% B from 0–50 min, 35–100% B from 50–58 min, and 100% B from 58–60 min) at a flow rate of 250 nL min^−1^. Mass spectrometry was performed in positive mode, with a full scan range of 300–1800 m/z, followed by MS/MS of the 10 most intense peaks in the linear ion trap. MS2 spectra were acquired at a resolution of 17 500 at 200 m/z, with dynamic exclusion set to 40 s. The resulting mass spectrometry data were processed and analyzed using Proteome Discoverer software to identify and quantify the proteomic profile of the femoral samples.

### Metabolomic Analysis of MLO‐Y4 Cells

MLO‐Y4 cells were harvested using a quenching solution composed of methanol, acetonitrile, and water in a ratio of 2:2:1. The samples were subjected to freeze‐thaw cycles in liquid nitrogen to facilitate cell disruption. Subsequently, 80 mg of each sample was mixed with 400 µL of a methanol/acetonitrile solution (1:1, v/v) and sonicated at a low temperature to extract metabolites. The samples were then centrifuged at high speed, and the supernatant was carefully collected and evaporated to dryness under a stream of nitrogen, yielding a powdery residue. This residue was reconstituted in a 50% acetonitrile solution and centrifuged again to obtain the metabolite‐containing supernatant, which was used for subsequent mass spectrometry analysis. The samples were analyzed using an ultra‐high‐performance liquid chromatography (UHPLC) system (1290 Infinity LC, Agilent Technologies) coupled to a QTRAP mass spectrometer (6500+, AB Sciex) at Shanghai Applied Protein Technology Co., Ltd. Chromatographic separation of the analytes was achieved using hydrophilic interaction liquid chromatography (HILIC) on a Waters UPLC BEH Amide column (2.1 mm × 100 mm, 1.7 µm). The column was maintained at 35 °C, and the injection volume was set at 2 µL. The mobile phases consisted of A: 90% water with 2 mM ammonium formate and 10% acetonitrile, and B: 0.4% formic acid in acetonitrile. A gradient elution program was applied (85% B from 0–1 min, 80% B from 3–4 min, 70% B at 6 min, 50% B from 10–15.5 min, and returning to 85% B from 15.6–23 min) at a flow rate of 300 µL min^−1^. Detection and quantification of the metabolites were performed by MS/MS in multiple reaction monitoring (MRM) mode. The MultiQuant software was used to extract the chromatographic peak areas and retention times for data analysis.

### LC‐MS/MS Analysis of SLC7A11‐Binding Proteins

Total protein was extracted from MLO‐Y4 cells. Subsequently, anti‐SLC7A11 antibodies and Pierce protein A/G magnetic beads were added to the cell lysate to selectively precipitate the SLC7A11 protein complex. The immune complexes were resolved by SDS‐PAGE, and the gel bands corresponding to the molecular weight of SLC7A11 were excised following Coomassie Brilliant Blue staining. The proteins within these bands were extracted, enzymatically digested into peptides, and desalted in preparation for LC‐MS/MS analysis, which was conducted by Suzhou PANOMIX Biomedical Tech Co., Ltd. (Suzhou, China). The peptides were separated using high‐performance liquid chromatography (HPLC) and subsequently introduced into a mass spectrometer. Mass spectrometry data acquisition was performed in data‐dependent acquisition (DDA) mode, which automatically selects precursor ions for MS/MS analysis based on their intensity. The resulting MS data were processed and analyzed using Proteome Discoverer software, and the MS data were searched against the Mus musculus database to identify the proteins.

### Molecular Dynamics Simulation and Docking Analysis

In the absence of experimental structures for the PSMD14‐SLC7A11 protein complex, this work employed the AlphaFold‐Multimer program to predict its structure.^[^ ^62^ ^]^ This work selected the most energetically favorable conformations for subsequent dynamic simulations and analysis. To form the PSMD14‐SLC7A11‐DEX ternary complex, this work conducted docking analysis. Specifically, this work used Autodock Tools to preprocess the PSMD14‐SLC7A11 protein complex and the DEX molecule, converting them into PDBQT formats. This work then employed Autodock Vina to dock the DEX molecule into the active site of the PSMD14‐SLC7A11 protein complex, thereby creating the PSMD14‐SLC7A11‐DEX ternary complex. For MD simulation, this work used the Desmond 2021.3 package to perform conventional MD simulations, aiming to explore the conformational changes within both the PSMD14‐SLC7A11 binary complex and the PSMD14‐SLC7A11‐DEX ternary complex. This work applied the opls2005 force field for protein parameterization and selected the TIP3P model for water. The protein complex was solvated in an octahedral water box, and the system's charge was neutralized by adding 0.150 M chloride and sodium ions. Initially, this work minimized the system's energy using the steepest descent method over 50 000 steps. Subsequently, this work constrained the positions of the heavy atoms during both the NVT (canonical ensemble) and NPT (isothermal‐isobaric ensemble) equilibration phases, each lasting 50 000 steps. The system temperature was maintained at 300 K and the pressure at 1 bar. After equilibration, this work conducted an unrestrained simulation for 100 ns, recording the energy and coordinates of the trajectory every 20 ps. Finally, ChimeraX and PyMOL were used to visualize the interaction patterns and animate the kinetic trajectories from the simulation data.

### Virtual Screening

Given the lack of an experimentally determined 3D structure for PSMD14, this work obtained the mouse PSMD14 structure (ID: AF‐O35593‐F1) from the AlphaFold database. Utilizing the Schrödinger software suite, this work employed the sitemap module to predict the most favorable binding site, designated as Site1, which achieved a high sitescore of 0.98. Site1, which is surrounded by key amino acids such as GLU134, ASP64, VAL25, LEU162, and others, was further processed using the Protein Preparation Wizard module of Schrödinger. The dimensions of the binding pocket were set to 20 Å × 20 Å × 20 Å (Figure S9A, Supporting Information). The MedChemExpress Bioactive Compounds Library Plus, containing 21 201 compounds (HY‐L001P), and the MedChemExpress 50K Diversity Library, containing 50 000 compounds (HY‐L901), were prepared for virtual screening after undergoing energy optimization using the LigPrep module of Schrödinger. These optimized compounds were then subjected to a Virtual Screening Workflow for molecular docking. Initially, all compounds were screened using Glide HTVS mode, and the top 15% of compounds based on scoring were advanced to Glide Standard Precision (SP) docking. Subsequently, the top 15% of compounds from Glide SP were further docked using Glide Extra Precision (XP). Ultimately, 20 molecules with the highest affinity, as indicated by a docking score greater than −7.6 kcal mol^−1^, were selected for subsequent experimental validation (Figure 6B). A higher absolute value of the predicted docking score signifies a stronger binding affinity. The 2D docking models were generated using PyMOL.

### Surface Plasmon Resonance Analysis

To investigate the binding affinity between PT (MedChemExpress) and recombinant mouse PSMD14 protein, SPR analysis was conducted using a BIAcore T200 instrument (GE Healthcare, USA). The PSMD14 antibody served as a positive control. The recombinant mouse PSMD14 protein was synthesized by MedChemExpress. SPR experiments were performed on a CM5 chip (Cytiva, MA, USA) with a running buffer of PBS. Binding and dissociation kinetics were measured at a flow rate of 30 µL min^−1^, with each ligand injection lasting 150 s, followed by a 5‐min flow of ligand‐free buffer for dissociation analysis. Sensorgrams were corrected for nonspecific binding by subtracting the negative control flow cell signal. Association and dissociation constants were determined by globally fitting the data to a 1:1 Langmuir binding model using BIAcore T200 Evaluation software (version 2.0, GE Healthcare).

### PSMD14 Activity Detection

PSMD14 activity was assessed using a Ubiquitin‐AMC assay kit (R&D Systems, MA, USA), adhering to established protocols.^[^ ^51^, ^63^ ^]^ Recombinant PSMD14 protein was incubated with various concentrations of PT in an assay buffer containing 40 mM Tris/HCl (pH 7.4), 5% glycerol, 0.005% Tween‐20, 1 mM dithiothreitol, and 0.05 mg mL^−1^ ovalbumin at 37 °C for 30 min. Ubiquitin‐AMC was then added to the reaction mixture and incubated for an additional 20 min. The fluorescence intensity, indicative of PSMD14 activity, was measured using a fluorescence spectrophotometer.

### Statistical Analysis

Data are presented as the mean ± standard deviation (SD) from a minimum of three independent experiments, as detailed in the figure legends. For pairwise comparisons, unpaired two‐tailed Student's *t*‐tests were used. When comparing more than two groups, one‐way analysis of variance (ANOVA) followed by Tukey's multiple comparisons test or two‐way ANOVA followed by Tukey's multiple comparisons test was employed to account for multiple comparisons. All statistical analyses were performed using Prism software (version 9.0, GraphPad, San Diego, CA, USA). A *P* value of less than 0.05 was considered statistically significant.

## Conflict of Interest

The authors declare no conflict of interest.

## Author Contributions

Y.S., Q.T., and S.S. contributed equally to this work. S.S. and L.Y. conceived and designed the study. Y.S., Q.T., and C.X. wrote the original draft. Q.T. and C.J. developed the methodology. Y.S., H.J., and H.M. performed experiments and investigations. W.N. and X.W. conducted formal statistical analyses. C.Z., X.P., and H.X. curated data and managed databases. D.Z. and H.X. developed software tools. L.Z., C.X., and G.X. validated experimental results. X.P. and A.W. provided critical resources. X.W. supervised project administration. Y.S., S.S., and C.J. prepared visualizations. A.W. and G.Z. oversaw the study and provided supervision. G.Z. acquired funding. Y.Q. reviewed and edited the manuscript. All authors read and approved the final manuscript.

## Supporting information



Supporting Information

Supplemental Table 1

Supplemental Table 2

Supplemental Table 3

Supplemental Table 4

## Data Availability

The data that support the findings of this study are available from the corresponding author upon reasonable request.
