# Supplementary material for: PSMD14 Stabilizes SLC7A11 to Ameliorate Glucocorticoid‐Induced Osteoporosis by Suppressing Osteocyte Ferroptosis
Source: Adv Sci (Weinh). 2025 May 30;12(31):e14902. doi: 10.1002/advs.202414902 (PMC12376700; doi:10.1002/advs.202414902)
Supplement: Supplementary file 1 — Supporting Information [file ADVS-12-e14902-s003.pdf]

ADVANCED SCIENCE
Open Access

Supporting Information

for *Adv. Sci.*, DOI 10.1002/advs.202414902

PSMD14 Stabilizes SLC7A11 to Ameliorate Glucocorticoid-Induced Osteoporosis by Suppressing Osteocyte Ferroptosis

Yifeng Shi, Qian Tang, Sunren Sheng, Hongyi Jiang, Chen Jin, Chencheng Zhou, Chenglong Xie, Lin Zheng, Di Zhang, Hui Xu, Cong Xu, Haiwei Ma, Guangheng Xiang, Wenfei Ni, Xiaoyun Pan, Lei Yang, Huazi Xu, Yu Qian, Aimin Wu*, Xiangyang Wang* and Gang Zheng**

Supplementary Information

PSMD14 Stabilizes SLC7A11 to Ameliorate Glucocorticoid-Induced Osteoporosis by Suppressing Osteocyte Ferroptosis

Yifeng Shi, Qian Tang, Sunren Sheng, Hongyi Jiang , Chen Jin, Chencheng Zhou, Chenglong Xie, Lin Zheng, Di Zhang, Hui Xu, Cong Xu, Haiwei Ma, Guangheng Xiang, Wenfei Ni, Xiaoyun Pan, Lei Yang, Huazi Xu, Yu Qian, Aimin Wu*, Xiangyang Wang*, Gang Zheng**

Figure S1-10
Table S1-8

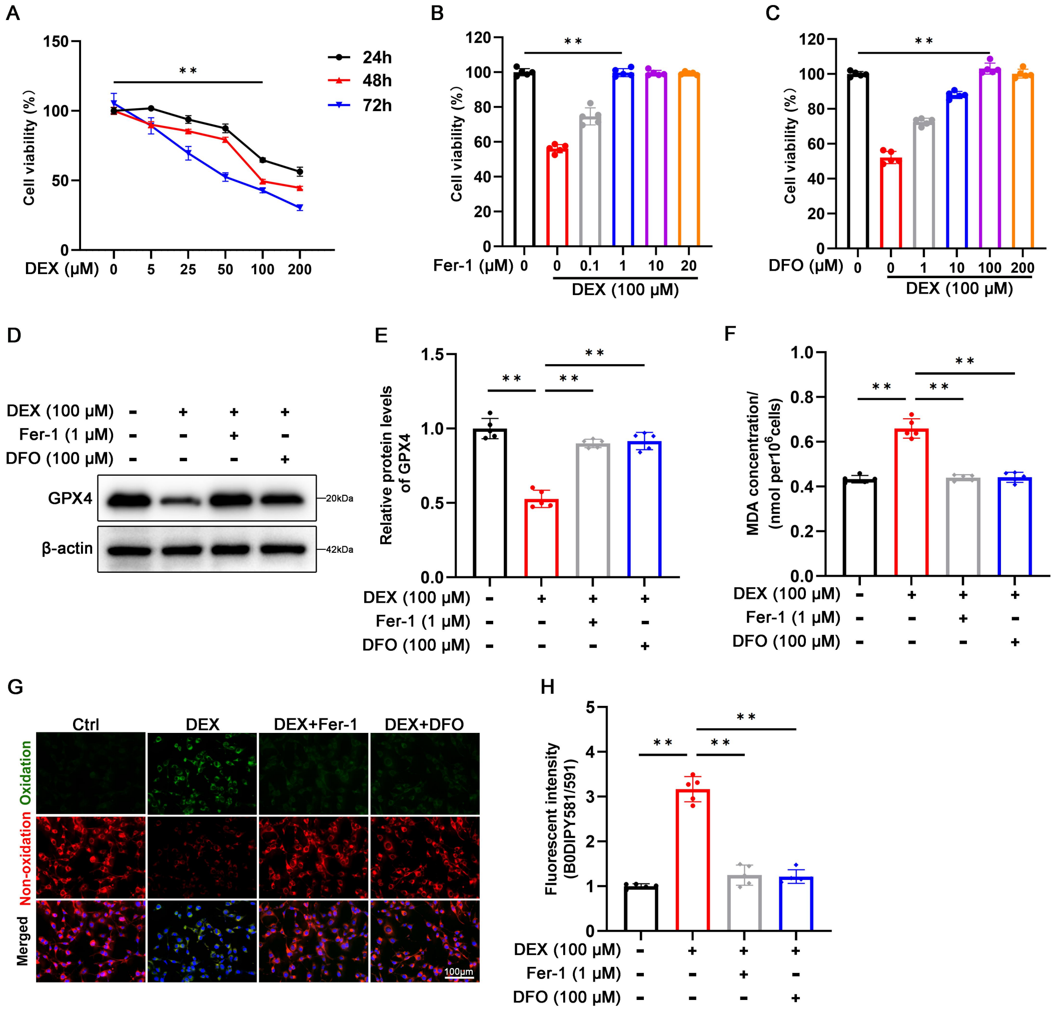

Figure S1. Dex initiated osteocyte ferroptosis *in vitro*.

(A) CCK-8 assay was performed on MLO-Y4 cells treated with different doses of DEX (5, 25, 50, 100 and 200 μM) for different time periods (24, 48 and 72 hours) to establish a glucocorticoid-stimulated osteocyte model *in vitro* (n = 5 per group). **(B)** CCK-8 assay was performed on MLO-Y4 cells treated with different doses of Fer-1 (0, 0.1, 1, 10, 20 μM) and DEX (100 μM) for 48 hours (n = 5 per group). **(C)** CCK-8 assay was performed on MLO-Y4 cells treated with different doses of DFO (0, 1, 10, 100, 200 μM) and DEX (100 μM) for 48 hours (n = 5 per group). **(D–H)** Western blot and quantitative analysis of GPX4 protein (D**,** E), MDA concentration detection (F) and C11-BODIPY 581/591 staining and quantitative analysis (G**,** H) were performed on MLO-Y4 cells treated with DEX (100 μM) supplemented with or wihtout ferroptosis inhibitors (Fer-1 1 μM or DFO 100 μM) for 48 hours (n = 5 per group). Data are expressed as mean ± SD, with biologically individual data points shown. *P* values were determined by one-way ANOVA test with Tukey's multiple comparisons (B,C,E,F,H) and two-way ANOVA with Tukey's multiple comparisons (A), * *P* < 0.05, ** *P* < 0.01.

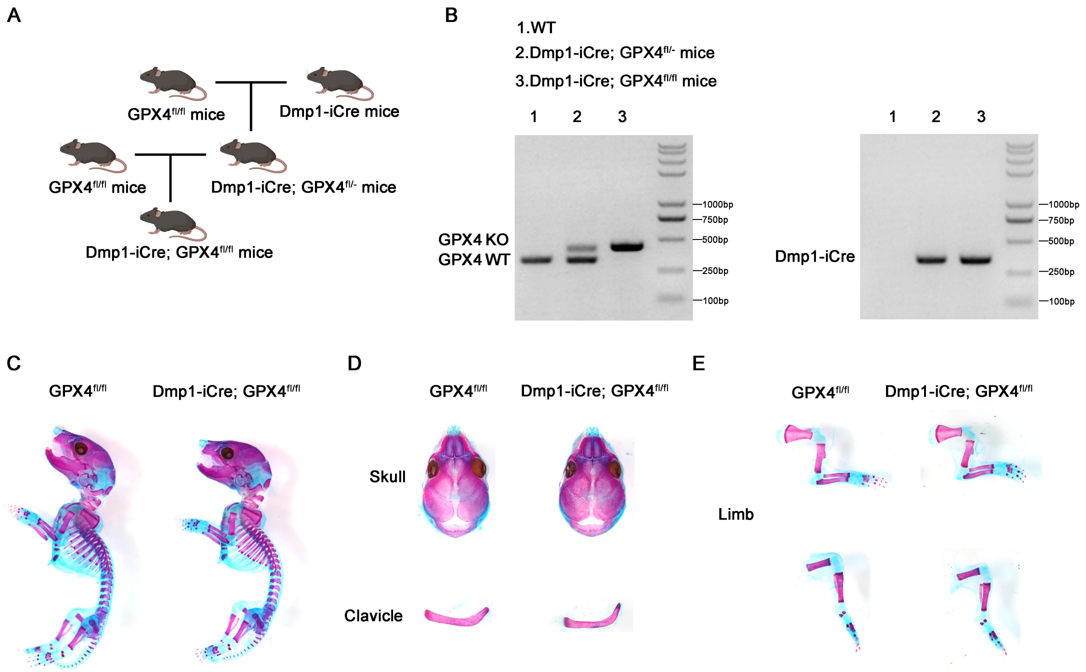

Figure S2. Construction of transgenic mice with osteocytic GPX4 knockout.
(A) Breeding strategy for generating the Dmp1-Cre; GPX4$^{fl/fl}$ cKO mice. **(B)** qPCR genotyping using tail DNA. GPX4^{fl} KO, 440bp; GPX4^{fl} WT, 335bp; Dmp1-Cre, 412bp. Primer sets are listed in Table S1. **(C–E)** Representative images of whole skeleton, skulls, clavicle, and limbs from male Dmp1-Cre and GPX4$^{fl/fl}$ mice newborns were double-stained with alcian blue and alizarin red S. The experiments were repeated three times independently with similar results.

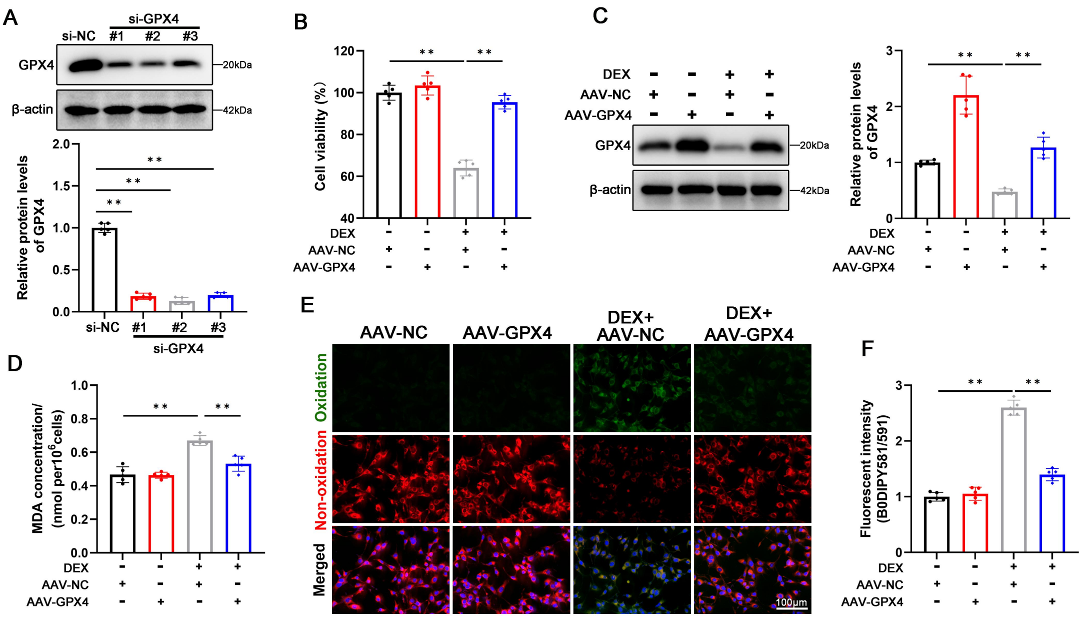

Figure S3. Overexpression of GPX4 inhibited DEX-mediated osteocyte ferroptosis.
(A) Western blot and quantitative analysis of GPX4 protein was performed on MLO-Y4 cells transfected with si-RNA (50 nM si-NC or si-GPX4 #1-3) (n = 5 per group). **(B–F)** CCK-8 assay (B), western blot and quantitative analysis of GPX4 proteins (C), MDA concentration detection (D), C11-BODIPY 581/591 staining and quantitative analysis (E, F) were performed on AAV-transfected MLO-Y4 cells. After AAV-NC/GPX4 transfection was completed, MLO-Y4 cells were treated with PBS or DEX (100 μM) for 48 hours (n = 5 per group). Data are expressed as mean ± SD, with biologically individual data points shown. *P* values were determined by one-way ANOVA test with Tukey's multiple comparisons (A–D,F), * $P < 0.05$, ** $P < 0.01$.

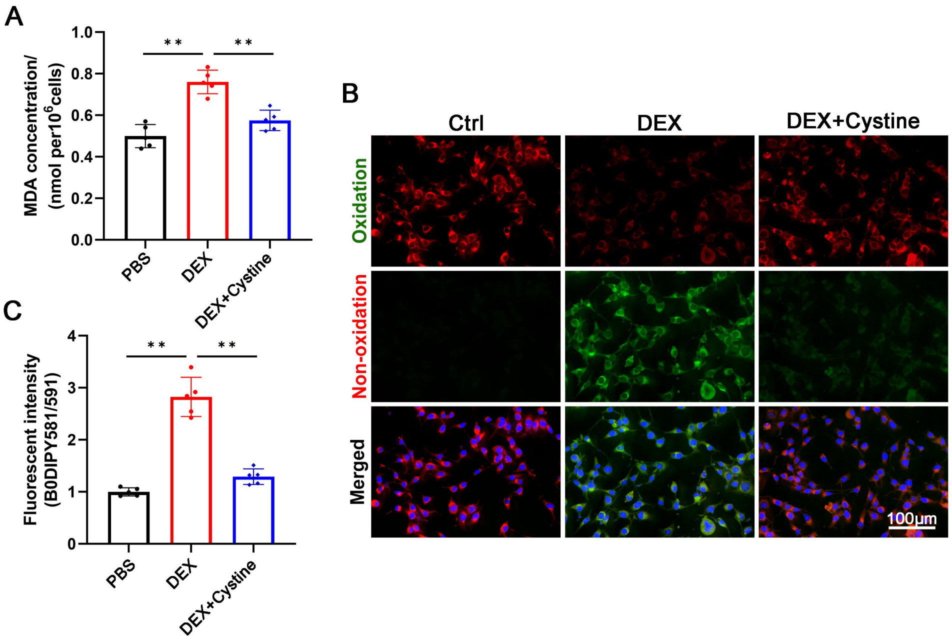

Figure S4. Exogenous cystine inhibited DEX-mediated osteocyte ferroptosis

(A–C) MDA concentration detection (A) and C11-BODIPY 581/591 staining and quantitative analysis (B**,** C) were performed on MLO-Y4 cells treated with DEX (100 μM) supplemented with or wihtout exogenous cystine (100 μM) for 48 hours (n = 5 per group). Data are expressed as mean ± SD, with biologically individual data points shown. *P* values were determined by one-way ANOVA test with Tukey's multiple comparisons (A,C), * $P < 0.05$, ** $P < 0.01$.

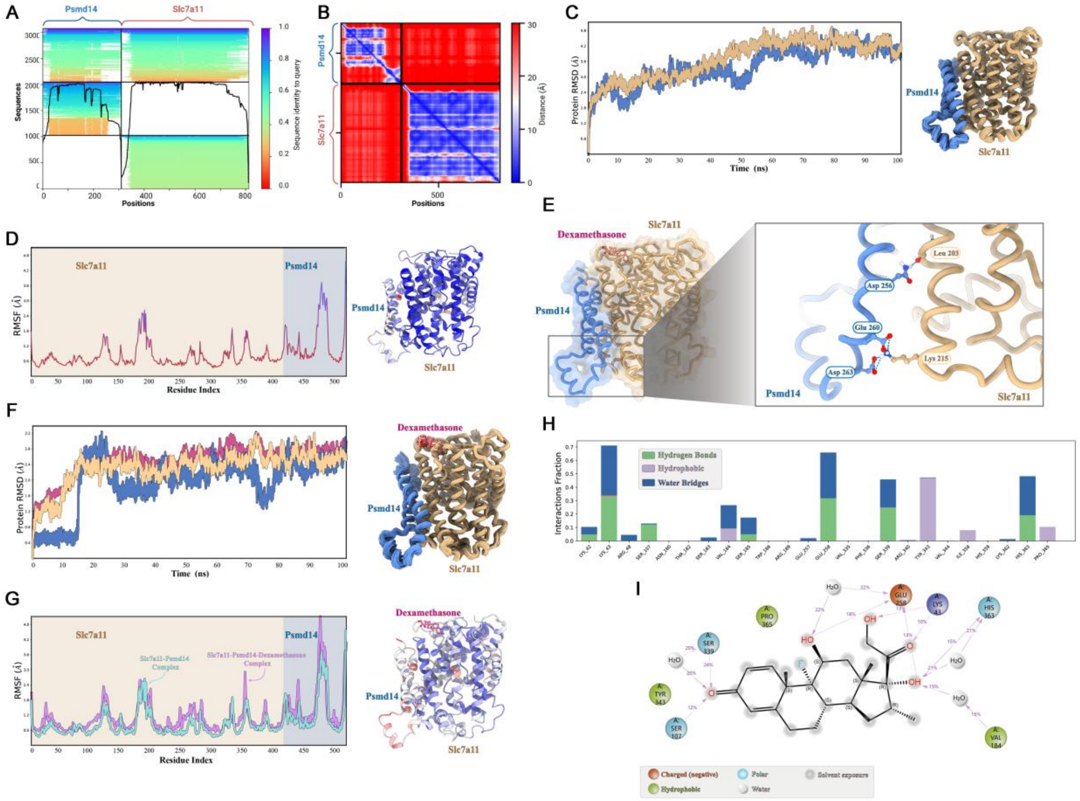

Figure S5. Molecular dynamics simulation and docking analysis of PSMD14-SLC7A11 and PSMD14-SLC7A11-DEX complexes.

(A) Multiple sequence alignment assay of the PSMD14-SLC7A11 complex. **(B)** Amino acid distance matrix assay of the PSMD14-SLC7A11 complex. **(C,D)** Root-mean-square deviation (RMSD, C) and Root-mean-square fluctuation (RMSF, D) of the PSMD14-SLC7A11 complex from molecular dynamics simulations. **(E)** Predicted ternary binding complex model of PSMD14-SLC7A11-DEX (left). The picture showed the hydrogen bonds in the protein interaction region and the corresponding amino acid residues (right). **(F,G)** RMSD (F) and RMSF (G) of the PSMD14-SLC7A11-DEX complex from molecular dynamics simulations. **(H,I)** Binding strength between amino acids in the SLC7A11 binding region and DEX molecules (H) and 2D schematic diagram of the interaction between DEX and SLC7A11 (I) based on the molecular dynamics simulations (90-100ns) of PSMD14-SLC7A11-DEX complex.

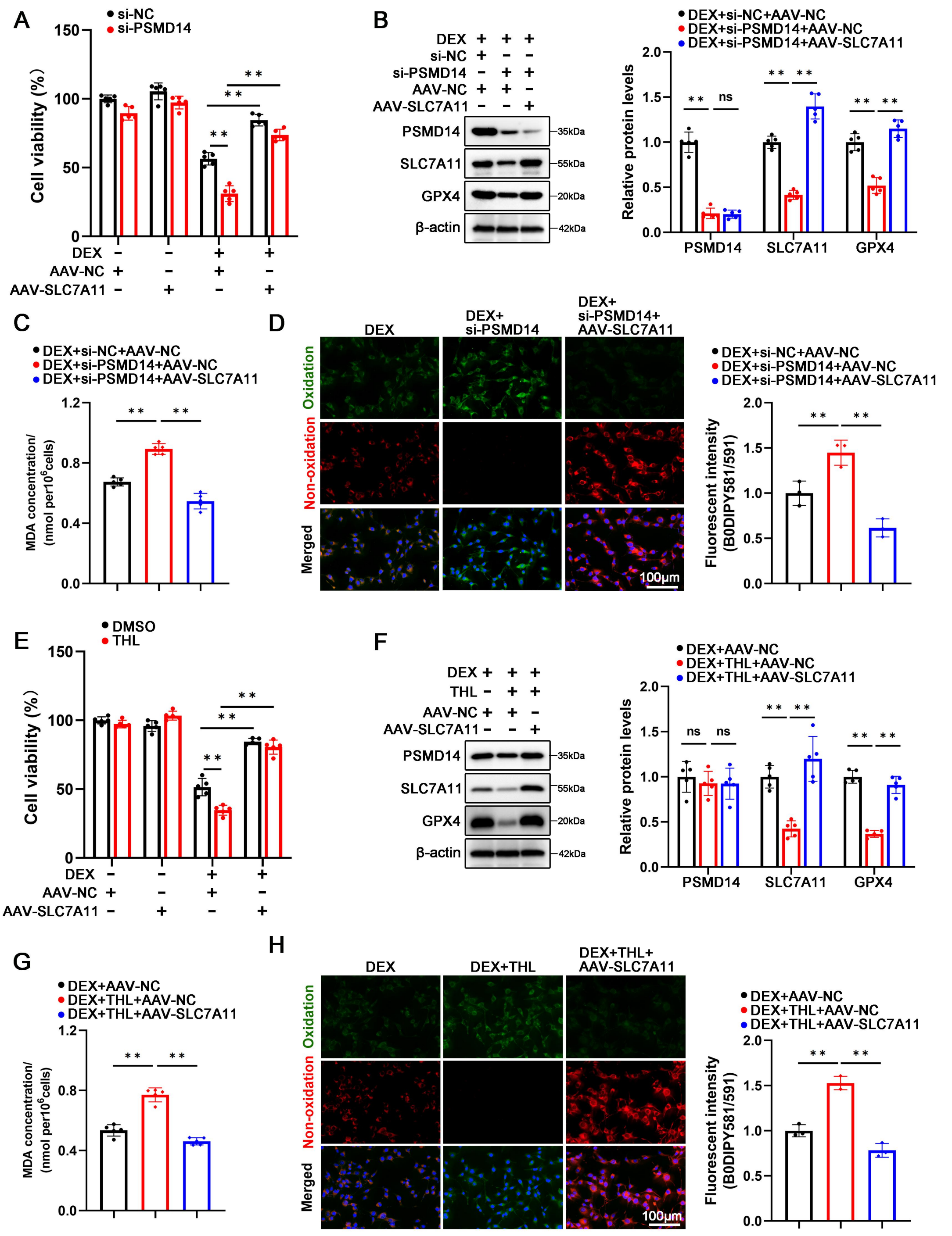

Figure S6. Inhibition of PSMD14 by siRNA or THL exacerbated DEX-mediated osteocyte ferroptosis *in vitro*.

(A–D) After si-PSMD14 and AAV-SLC7A11 transfection were completed, MLO-Y4 cells were treated with PBS or DEX (100 μM) for 48 hours. CCK-8 assay (A), western blot and quantitative analysis of PSMD14, SLC7A11 and GPX4 proteins (B), MDA concentration detection (C) and C11-BODIPY 581/591 staining (D) were performed (n = 5 per group). **(E–H)** After AAV-SLC7A11 transfection were completed, MLO-Y4 cells were treated with DEX (100 μM) and/or THL (2 μM) for 48 hours. CCK-8 assay (E), western blot and quantitative analysis of PSMD14, SLC7A11 and GPX4 proteins (F), MDA concentration detection (G) and C11-BODIPY

581/591 staining (H) were performed (n = 5 per group). Data are expressed as mean ± SD, with biologically individual data points shown. *P* values were determined by two-way ANOVA test with Tukey's multiple comparisons (A,B,E,F) and one-way ANOVA test with Tukey's multiple comparisons (C,D,G,H), * $P < 0.05$, ** $P < 0.01$.

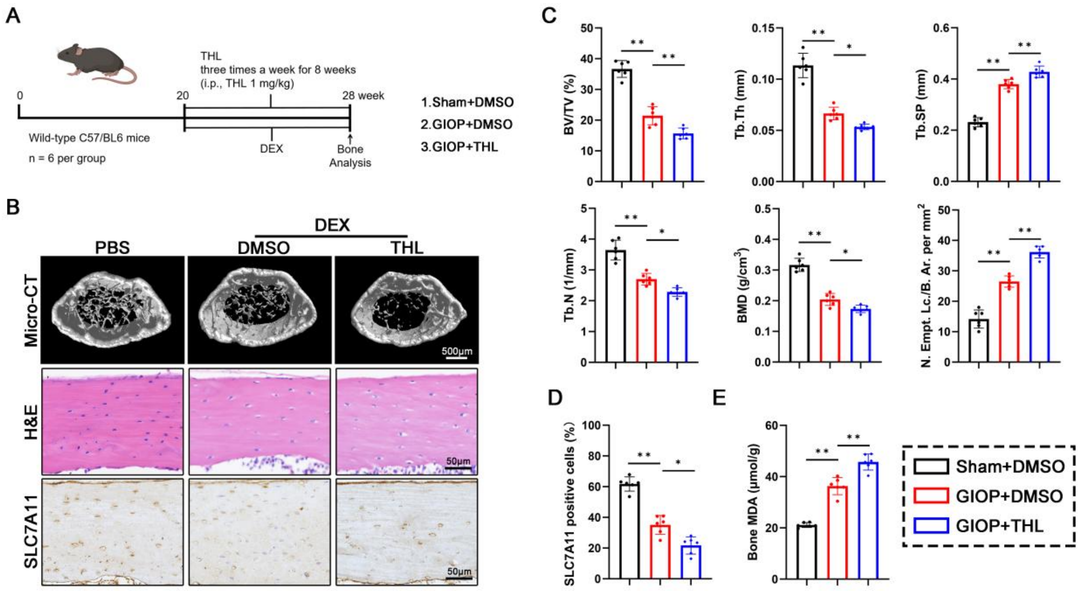

Figure S7. THL promoted DEX-mediated ferroptosis and bone loss.

(A) Schematic showing the experimental protocol for 8-weeks of THL injections in GIOP mice. **(B)** Micro-CT 3D restruction, H&E staining, and SLC7A11 histochemical staining of the distal femur of mice in each group. The processing details of each group are shown in Figure 7A. **(C)** Distal femur BV/TV, Tb.Th, Tb.Sp, Tb.N, and BMD of mice in each group were measured by micro-CT (n = 6 per group). Quantitative analysis of the empty lacunae in cortical bone (Number of empty lacunae with respect to bone area, N. Empt. Lc./B. Ar. per mm^2) based on H&E staining (n = 6 per group). **(D)** Quantification of SLC7A11-positive osteocytes in mouse cortical femurs based on IHC staining (n = 6 per group). **(E)** MDA content in mouse tibia tissue (n = 6 per group). Data are expressed as mean ± SD, with biologically individual data points shown. *P* values were determined by one-way ANOVA test with Tukey's multiple comparisons (C–E), * $P < 0.05$, ** $P < 0.01$.

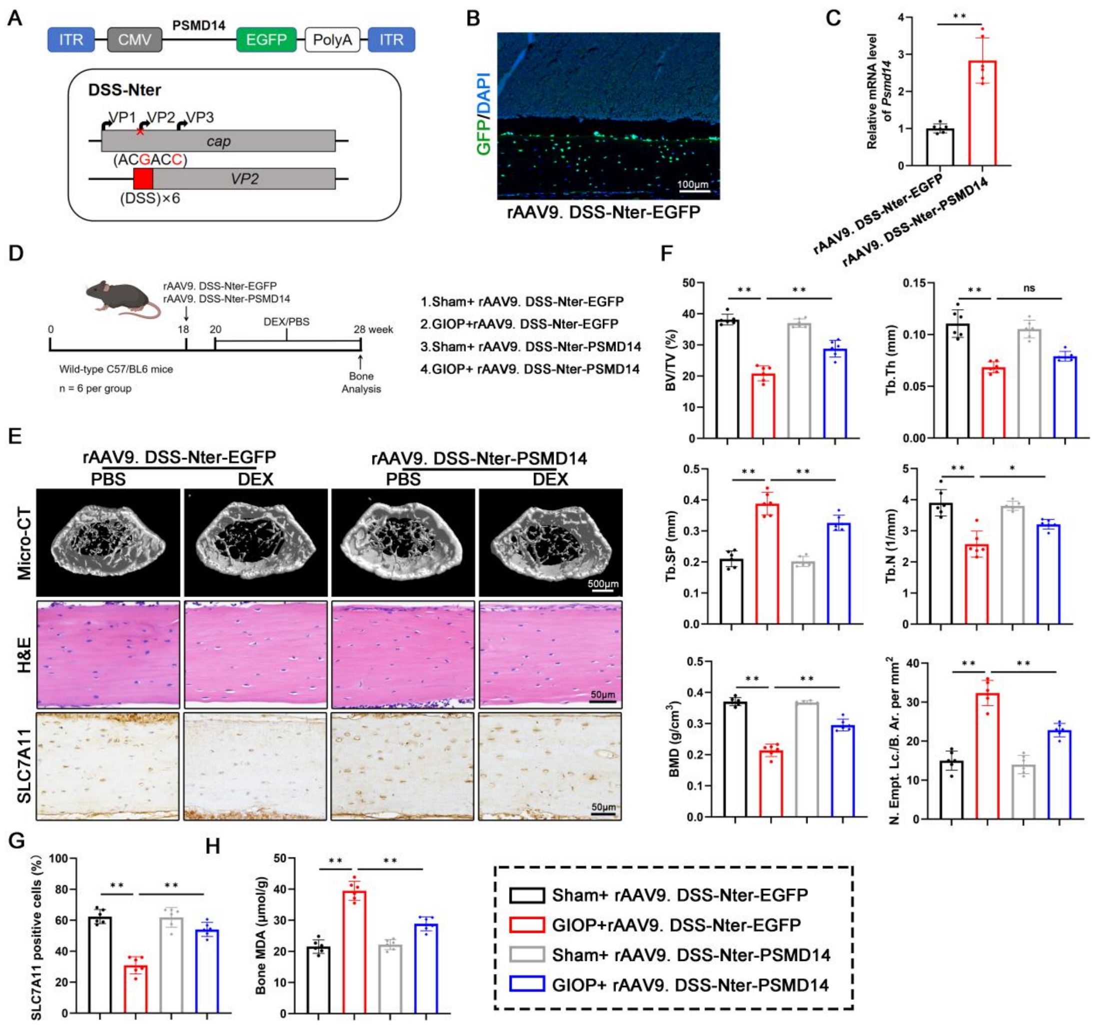

Figure S8. Targeted overexpression of PSMD14 *via* rAAV9 rescued DEX-mediated ferroptosis and osteoporosis.

(A) The bone-targeting rAAV9-EGFP construct was designed with a CMV enhancer/chicken β-actin promoter (CB), the *Psmd14* gene, an *Egfp* reporter gene (EGFP), a β-globin polyA sequence (PA), and inverted terminal repeats (ITRs). The bone-targeting peptide motif (DSS, red) was inserted into the AAV9 capsid at the N-terminus of AAV9-VP2 (DSS-Nter). Cap: capsid proteins. **(B)** Representative immunofluorescence images of EGFP in mice after intravenous injection of rAAV9.DSS-Nter-EGFP at a dose of 4×10^{11} genome particles for 2 months. **(C)** *Psmd14* mRNA levels in the tibia of AAV-treated mice for 2 months were assessed by qPCR. **(D)** Schematic showing the experimental protocol for rAAV9-DSS-Nter-PSMD14 tail vein injection in GIOP mice. **(E)** Micro-CT 3D restruction, H&E staining, and SLC7A11 histochemical staining of the distal femur of mice in each group. The processing details of each group are shown in Figure S8A. **(F)** Distal femur BV/TV, Tb.Th, Tb.Sp, Tb.N, and BMD of mice in each group were measured by micro-CT (n = 6 per group). Quantitative analysis of the empty lacunae in cortical bone (Number of empty lacunae with respect to bone area, N. Empt. Lc./B. Ar. per mm^2) based on H&E staining (n = 6 per group). **(G)** Quantification of SLC7A11-positive osteocytes in mouse cortical femurs based on IHC staining (n = 6 per group). **(H)** MDA content in mouse tibia tissue (n = 6 per group). Data are expressed as mean ± SD, with biologically individual data points shown.

P values were determined by unpaired two-tailed Student's t test (C) and two-way ANOVA test with Tukey's multiple comparisons (F–H), * $P < 0.05$, ** $P < 0.01$.

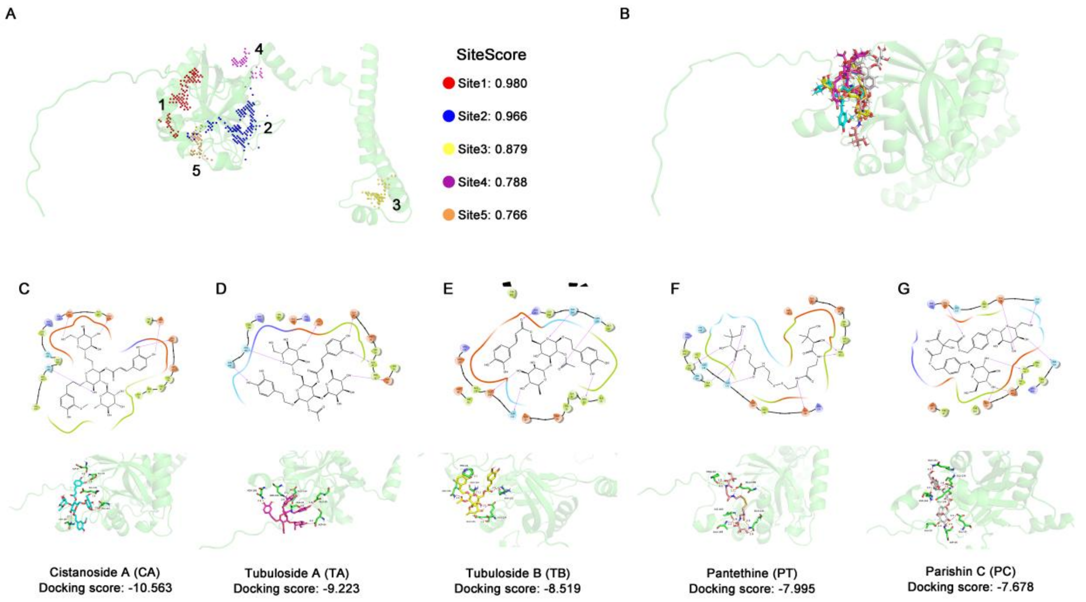

Figure S9. Molecular docking analysis of candidate compounds with PSMD14.
(A) The sitemap module within the Schrödinger software suite was utilized to predict the most favorable binding site (Site 1-5, Sitecore from high to low). **(B)** Representative docking images of PSMD14 and five drug candidates (CA, TA, TB, PT and PC). **(C–G)** The binding modes of the five compounds with PSMD14 protein were depicted in 2D (top) and 3D (bottom) illustrations. CA (C), TA (D), TB (E), PT (F) and PC (G).

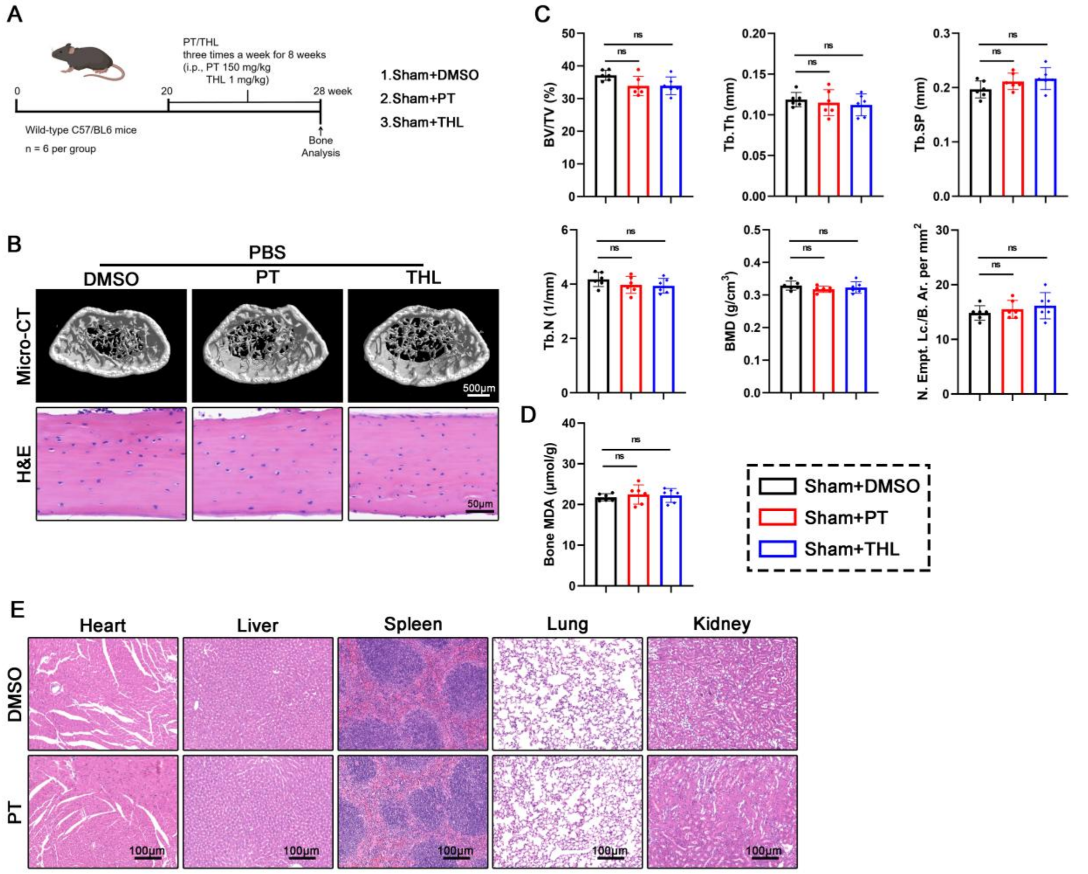

Figure S10. Effects of PT and THL on osteocytes and bone mass under physiological conditions.

(A) Schematic showing the experimental protocol for 8-weeks of PT or THL injections in GIOP mice. **(B)** Micro-CT 3D restruction and H&E staining of the distal femur of mice in each group. The processing details of each group are shown in Figure S10A. **(C)** Distal femur BV/TV, Tb.Th, Tb.Sp, Tb.N, and BMD of mice in each group were measured by micro-CT (n = 6 per group). Quantitative analysis of the empty lacunae in cortical bone (Number of empty lacunae with respect to bone area, N. Empt. Lc./B. Ar. per mm^2) based on H&E staining (n = 6 per group). **(D)** MDA content in mouse tibia tissue (n = 6 per group). **(E)** H&E staining of indicated organs. Data are expressed as mean ± SD, with biologically individual data points shown. *P* values were determined by ordinary one-way ANOVA test with Tukey's multiple comparisons (C,D), ns, $P > 0.05$, * $P < 0.05$, ** $P < 0.01$.

Table S1. Proteomic data of femur of control and GIOP mice

Table S2. Metabolomics analysis data of MLO-Y4 cells

Table S3. LC-MS/MS analysis data of SLC7A11-bound deubiquitinase in MLO-Y4 cells

Table S4. The top 100 compounds with the highest docking scores from virtual screening

Table S5. The sequence of primers used in PCR genotyping

Gene name	Forward	Reverse
Cre	GGGCAGTCTGGTACTTCCAAGCT	AGCTGCACCATCAACATGCC
Gpx4	GTACTGCAACAGCTCCGAGTTC	ACTTATCCAGGCAGACCATGTG

Table S6. The sequence of siRNA

siRNA	Sense	Antisense
si-Negative Control	UUCUCCGAACGUGUCACGUTT	ACGUGACACGUUCGGAGAATT
si-*Gpx4* #1	GGAUGAAAGUCCAGCCCAATT	UUGGGCUGGACUUUCAUCCTT
si-*Gpx4* #2	CCAAGUUUCUCAUUGAUAATT	UUAUCAAUGAGAAACUUGGTT
si-*Gpx4* #3	GCUGAGUGUGGUUUACGAATT	UUCGUAAACCACACUCAGCTT
si-*Psmd14* #1	GGACAUGAACCAAGACAAATT	UUUGUCUUGGUUCAUGUCCTT
si-*Psmd14* #2	GGAACAGAAGAUGCUGUUATT	UAACAGCAUCUUCUGUUCCTT
si-*Psmd14* #3	GCCUUGCUAAAGAUGUUAATT	UUAACAUCUUUAGCAAGGCTT

Table S7. Antibody information

Antibody	Sources	Catalog#	Dilution
Anti-GPX4	Abcam	ab125066	IHC (1:200)<br>WB (1:1000)
Anti-SLC7A11	Abcam	ab307601	IHC (1:100)<br>WB (1:1000)<br>IP (1:100)
Anti-β-actin	Proteintech	66009-1-Ig	WB (1:3000)
Anti-Ub	Proteintech	10201-2-AP	WB (1:1000)
Anti-PSMD14	Cell Signaling Technology	#4197	WB (1:1000)<br>IP (1:100)
Anti-PSMD14	Abcam	ab109123	IHC (1:100)
Anti-OTUB1	Cell Signaling Technology	#3783	WB (1:1000)
Anti-c-Myc	Cell Signaling Technology	#18583	WB (1:1000)<br>IP (1:100)
Anti-Flag	Cell Signaling Technology	#14793	WB (1:1000)<br>IP (1:50)
Anti-HA	Proteintech	51064-2-AP	WB (1:1000)
HRP-conjugated Goat Anti-Rabbit IgG(H+L)	Proteintech	SA00001-2	WB (1:3000)
HRP-conjugated Goat Anti-Mouse IgG(H+L)	Proteintech	SA00001-1	WB (1:3000)

Table S8. The sequence of primers used in RT–qPCR

Gene name	Forward	Reverse
Slc7a11	AATACGGAGCCTTCCACGAG	TTGCTATCACCGACTGGCTC
Psmd14	TGGCCGCAATGTTGGATACT	AAGCGAGTGCACAACTGAGA
Actb	ACAGCAGTTGGTTGGAGCAA	ACGCGACCATCCTCCTCTTA
